# Supplementary material for: Generation, expansion, gene delivery, and single-cell profiling in rhesus macaque plasma B cells
Source: Cell Rep Methods. 2024 Oct 14;4(10):100878. doi: 10.1016/j.crmeth.2024.100878 (PMC11573788; doi:10.1016/j.crmeth.2024.100878)
Supplement: Document S1. Figures S1–S7 and Tables S1 and S2 [file mmc1.pdf]

Cell Reports Methods, Volume *4*

Supplemental information

Generation, expansion, gene delivery, and single-cell profiling in rhesus macaque plasma B cells

Rene Yu-Hong Cheng, Anna E. Helmers, Shannon Kreuser, Noelle Dahl, Yuchi Honaker, Christina Lopez, David J. Rawlings, and Richard G. James

Supplemental

Table S1: Staining Panels. Related to Figures 1, 5, and 7.

**Panel 1: Day 0 Purity assessment**					
**Antigen**	Fluorophore	Clone	Vendor	Cat#	Dilution
**Live Dead**	AF350	N/A	Invitrogen	A10168	1:1000
**CD20**	PerCP-Cy5.5	L27	BD Bioscience	340955	1:20
**CD14**	APC-Cy7	MoP9	BD Bioscience	557831	1:200
**CD56**	PE	MY31	BD Bioscience	347747	1:200
**CD4**	BV605	L200	BD Bioscience	562843	1:200
**CD3**	A700	SP34	BD Bioscience	557917	1:200
**Panel 2: Phenotyping and transduction efficiency panel**					
**Antigen**	Fluor	Clone	Vendor	Cat#	Dilution
**Live Dead**	AF350	N/A	Invitrogen	A10168	1:1000
**CD20**	PerCP-Cy5.5	L27	BD Bioscience	340955	1:100
**CD14**	APC-Cy7	MoP9	BD Bioscience	557831	1:200
**CD31**	PE	WM59	BD Bioscience	555446	1:100
**CD38**	mFluor450	OKT10	Caprico Biotechnologies	1008144	1:100
**CD3**	AF700	SP34	BD Bioscience	557917	1:200
**Transduction**	GFP	--	---		---
**Panel 3: Phenotyping and intracellular Ig staining**					
**Antigen**	Fluor	Clone	Vendor	Cat#	Dilution
**Live Dead**	AF350	N/A	Invitrogen	A10168	1:1000
**CD20**	PerCP-Cy5.5	L27	BD Bioscience	340955	1:20
**CD14**	APC-Cy7	MoP9	BD Bioscience	557831	1:200
**CD138**	PE	DL-101	Biolegend	352306	1:100

**CD38**	mFluor450	OKT10	Caprico Biotechnologies	1008144	1:50
**CD38**	FITC	OKT10	Caprico Biotechnologies	100815	1:200
**CD3**	APC-Cy7	SP34	BD Bioscience	557917	1:200
**CD31**	BV605	WM59	Biolegend	303122	1:100
**CD59**	APC	p282	Biolegend	304712	1:50
**CD79A**	BV421	HM47	BD Bioscience	BDB566225	1:200
**IgG-ic**	AF700	G18-145	BD Bioscience	561296	1:200
**IgM-ic**	FITC	G20-127	BD Bioscience	555782	1:200

Table S2: AAV transduction with different titers. Related to Figure 7.

		DAY 5		
		698	A17104	relative titer
	no AAV	1.55	0.58	0
**1**	scAAV1	9.41	2.33	0.664063
**2**	AAV2	18.4	9.41	1
**3**	AAV2.5	11.2	6.36	0.46875
**4**	scAAV6	9.46	2.09	0.086328
**5**	AAVD-J	22	9.92	1.757813
**6**	AAV5	28.4	12.3	4.84375
**7**	AAV8	7.48	1.75	2.402344
**8**	AAV9	3.96	0.99	2.773438
**9**	AAVRh.8	1.1	0.36	0.332031
**10**	AAV11cap11	0.91	0.34	0.214844
**11**	AAVcap11	0.94	0.4	0.46875
**12**	AAV HSC7	1.8	0.59	0.992188
**13**	AAV1110 pLT AAV Help (AAV2) Y444F, Y500F, Y730F	16.2	6.95	0.5
		DAY 7		
		698	A17104	relative titer
	no AAV	2.42	1.05	0
**1**	scAAV1	5.91	2.34	0.664063
**2**	AAV2	26.5	23.9	1
**3**	AAV2.5	14.6	11.3	0.46875
**4**	scAAV6	6.74	1.82	0.086328
**5**	AAVD-J	33.7	23.2	1.757813
**6**	AAV5	26.2	14.5	4.84375
**7**	AAV8	5.18	2.18	2.402344
**8**	AAV9	3.2	1.9	2.773438

**13**	AAV1110 pLT AAV Help (AAV2) Y444F, Y500F, Y730F	19	13.7	0.332031
		DAY 10		
		698	A17104	relative titer
	no AAV	7.27	1.63	0
**1**	scAAV1	3.28	1.37	0.664063
**2**	AAV2	21.5	21.1	1
**3**	AAV2.5	10.4	8.34	0.46875
**4**	scAAV6	3.78	1.3	0.086328
**5**	AAVD-J	35.6	26.4	1.757813
**6**	AAV5	22.9	14.5	4.84375
**7**	AAV8	4.27	2.11	2.402344
**8**	AAV9	2.92	1.58	2.773438
**13**	AAV1110 pLT AAV Help (AAV2) Y444F, Y500F, Y730F	12.5	10.5	0.332031

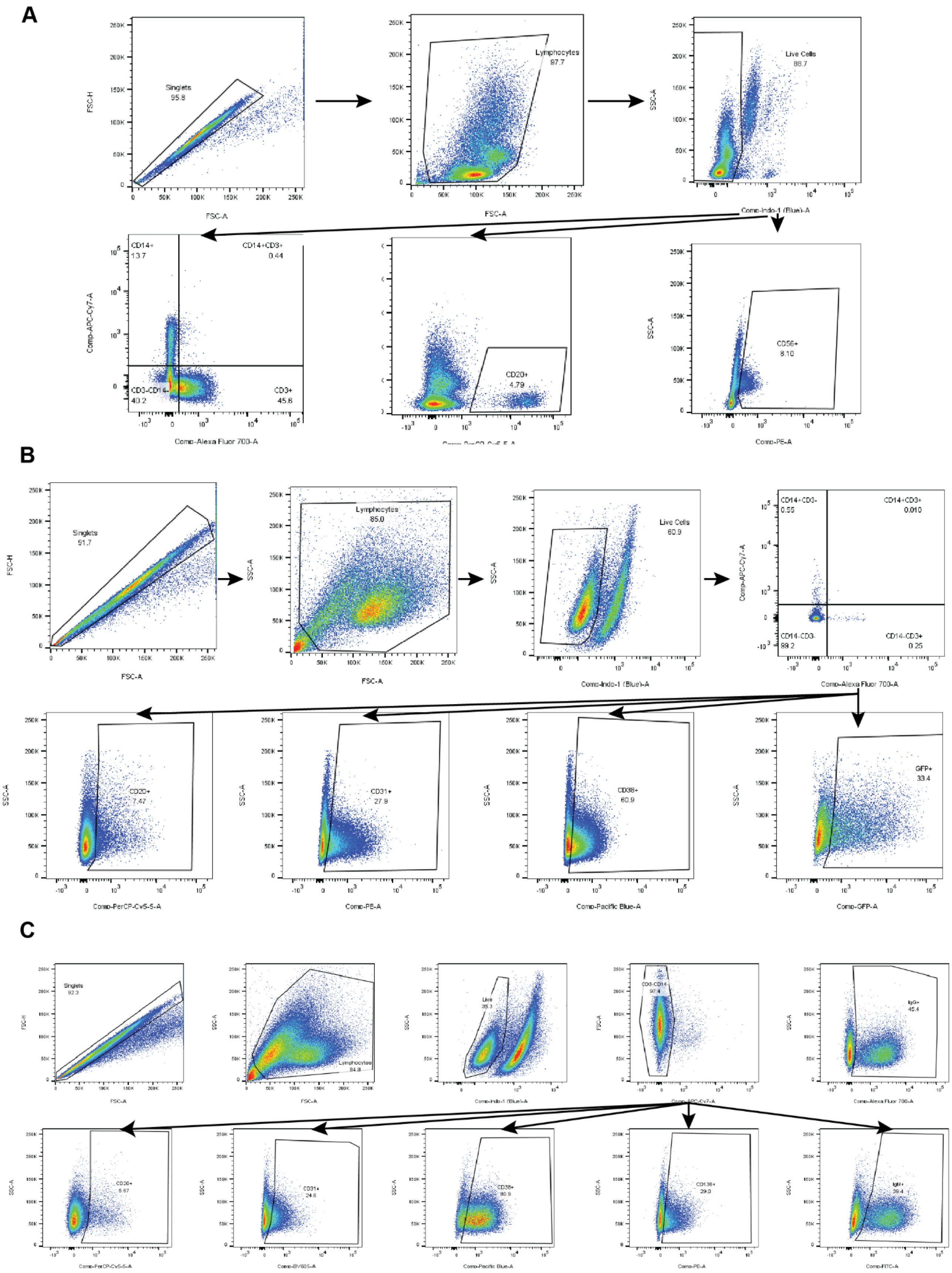
A
Singlets
95.8
FSC-H
FSC-A
Lymphocytes
97.7
Live Cells
88.7
SSC-A
Comp-Indo-1 (Blue)-A
CD14+
13.7
CD14+CD3+
0.44
CD3-CD14-
40.2
CD3+
45.6
Comp-APC-Cy7-A
Comp-Alexa Fluor 700-A
CD20+
4.79
CD56+
8.10
Comp-PE-A
B
Singlets
91.7
Lymphocytes
85.0
Live Cells
60.9
CD14+CD3-
0.55
CD14+CD3+
0.010
CD14-CD3-
99.2
CD14-CD3+
0.25
CD20+
7.47
CD31+
27.9
CD38+
60.9
GFP+
33.4
Comp-PerCP-Cy5-5-A
Comp-Pacific Blue-A
Comp-GFP-A
C
Singlets
92.3
Lymphocytes
84.8
Live
35.3
CD3-CD14-
97.4
IgG+
45.4
CD20+
5.67
CD31+
24.6
CD38+
80.9
CD138+
29.0
IgM+
39.4
Comp-BV605-A
Comp-FITC-A

Figure S1. Flow cytometry gating strategy. Related to Figures 1, 5, and 7. A. Gating scheme for flow cytometry Panel 1 (Table S1) to determine purity at the time of isolation. **B.** Gating scheme for flow cytometry Panel 2 (Table S1) to determine B cell phenotype and transduction efficiency through GFP expression. **C.** Gating scheme for flow cytometry Panel 3 (Table S1) to determine B cell phenotype and intracellular Ig production.

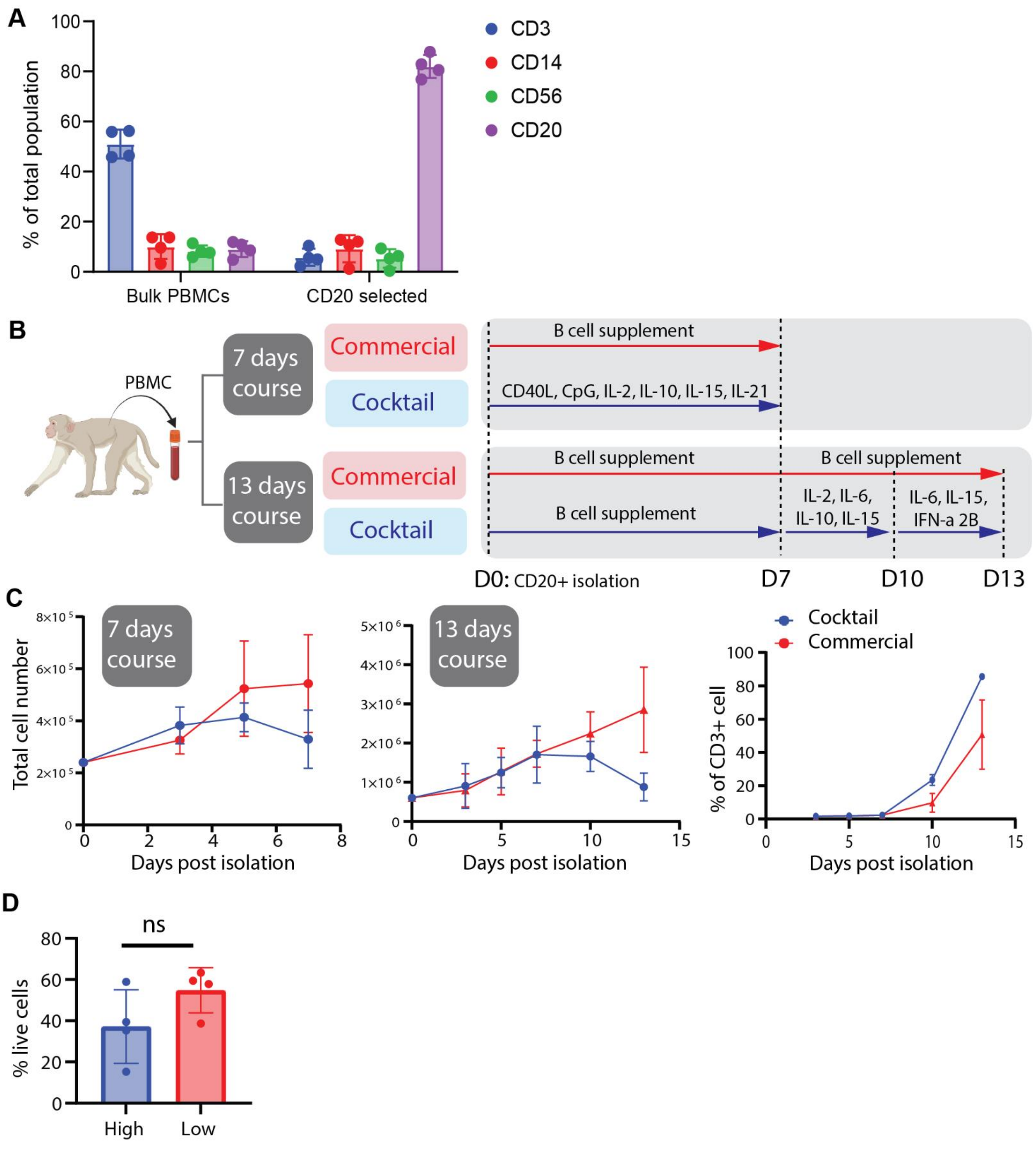

Figure S2. Method of ex vivo expansion and differentiated NHP PCs. Related to Figure 1. A. Following isolation of CD20+ Rhesus B cells from PBMCs, we assessed cell purity via flow cytometry using the indicated antibodies (4 donors, n=4). **B.** Schematic workflow of monkey *ex vivo* differentiated PC generation with two different time course and two different conditions. **C.** CD20+ NHP B cells were cultured for 7 days/13 days with defined cocktail or commercial expansion medium (1.5-1x10^6 cells/mL) (3 donors, n=3), Total cells were counted on days 3, 5, and 7 (left and middle). Percentage of contaminating

T cells cells (CD3+, left). **D.** To assess the impact of cell density, CD20+ NHP B cells were cultured in the commercial B cell medium for 7 days (Plated and maintained $1.5x10^6$ cells/mL versus plated at $2.5x10^5$ cells/mL and maintained at $1x10^5$ cells/mL; 4 donors, n=3). At day 7 following isolation, the percent viability was analyzed using flow cytometry. Data presented as individual data points and error bar represent standard deviation.

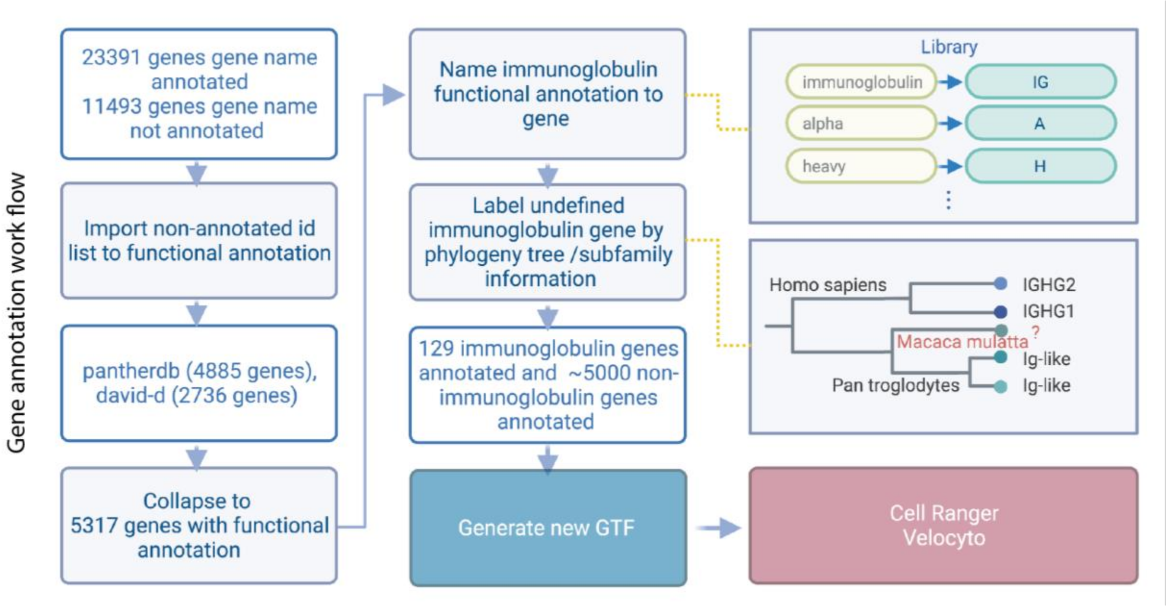

Figure S3. Monkey (Macaca mulatta) gene annotation workflow. Related to Figure 1. Gene annotation workflow. (GTF: Gene transfer fomat, Macaca_mulatta.Mmul_10.104.chr.gtf, generated new GTF: genes.gtf)

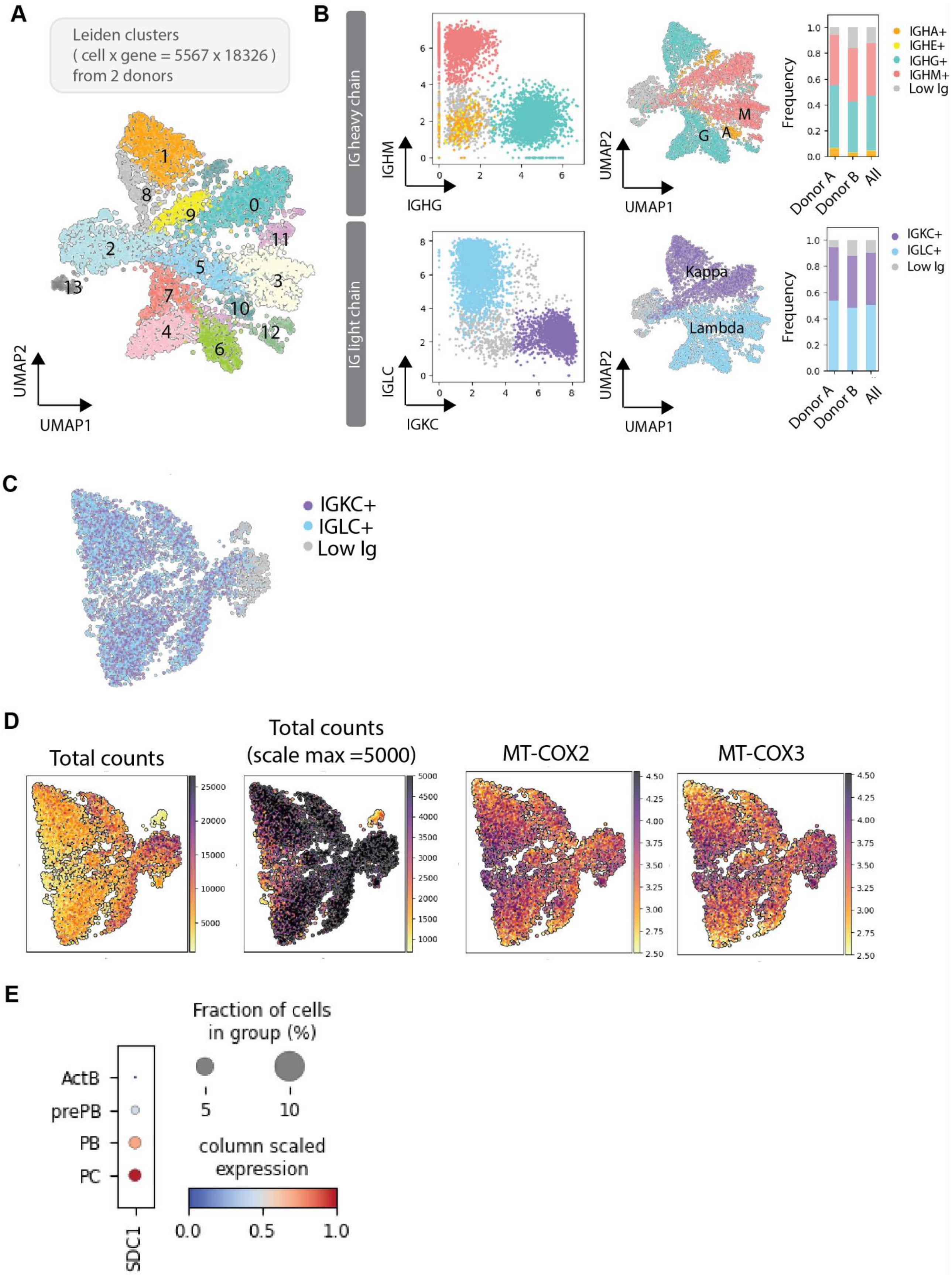
A
Leiden clusters
(cell x gene = 5567 x 18326)
from 2 donors
0
1
2
3
4
5
6
7
8
9
10
11
12
13
UMAP2
UMAP1
B
IG heavy chain
IGHM
IGHG
UMAP2
UMAP1
M
G
A
Frequency
Donor A
Donor B
All
IGHA+
IGHE+
IGHG+
IGHM+
Low Ig
IG light chain
IGLC
IGKC
Kappa
Lambda
IGKC+
IGLC+
Low Ig
C
IGKC+
IGLC+
Low Ig
D
Total counts
Total counts
(scale max =5000)
MT-COX2
MT-COX3
E
ActB
prePB
PB
PC
SDC1
Fraction of cells
in group (%)
5
10
column scaled
expression
0.0
0.5
1.0

Figure S4. Monkey *ex vivo* differentiated PCs subsets classification and differential expression analysis for PCs. Related to Figures 1, 2, and 3. A. Single cell graph UMAP dimension-reduction projection of day13 B cells (n = 5567) from two biological replicates. **B.** Classification of immunoglobulin heavy chain (upper panel) and light chain (lower panel). UMAP projection of the subclass of IGHG+, IGHA+, IGHM+ cells (upper panel) and IGKC+, IGLC+ cells (lower panel), and bar graph represent the percentage of subclasses from two individual donor. **C.** UMAP projection of IGKC+, IGLC+ cells in LC-independent clusters. **D.** Heatmap showing total count and expression of representative MT genes in LC-independent clusters. **E.** Dotplot visualization of *ex vivo* differentiated monkey PCs: subsets are listed on y-axis and SDC1 (CD138) gene on x-axis.

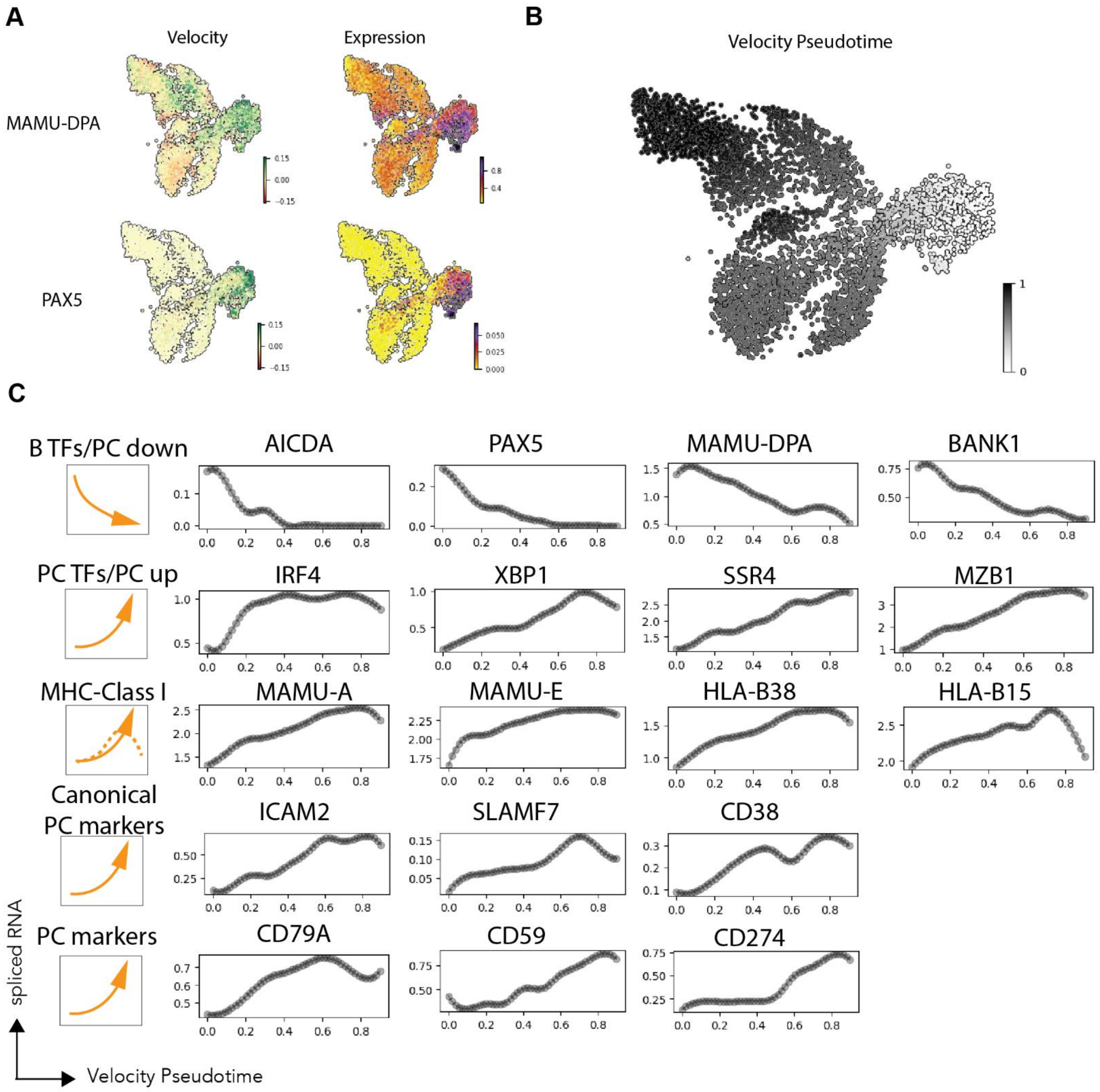

D

	fit_diff_kinetics	fit_pval_kinetics
**EZH2**	ActB	1.70e-08
**TUBA1A**	ActB	1.97e-06
**BIRC3**	prePB	1.08e-03
**MAN1A1**	mPC	1.56e-04
**FUT8**	aPC	8.95e-03
**FNDC3A**	aPC	6.73e-04
**IGF1**	gPC	3.10e-03
**CD274**	gPC	2.30e-06

Figure S5. RNA velocity of immunoglobulin and kinetic differentiation analysis. Related to Figure 4. A. Heatmap of indicated gene velocity and RNA expression level superimposed on to UMAP. **B.** Heatmap of velocity pseudotime superimposed on to UMAP. **C.** Scatter plot of indicated genes with velocity pseudotime and mean of spliced mRNA in every 0.1 pseudotime. **D.** scVelo kinetic differentiation analysis.

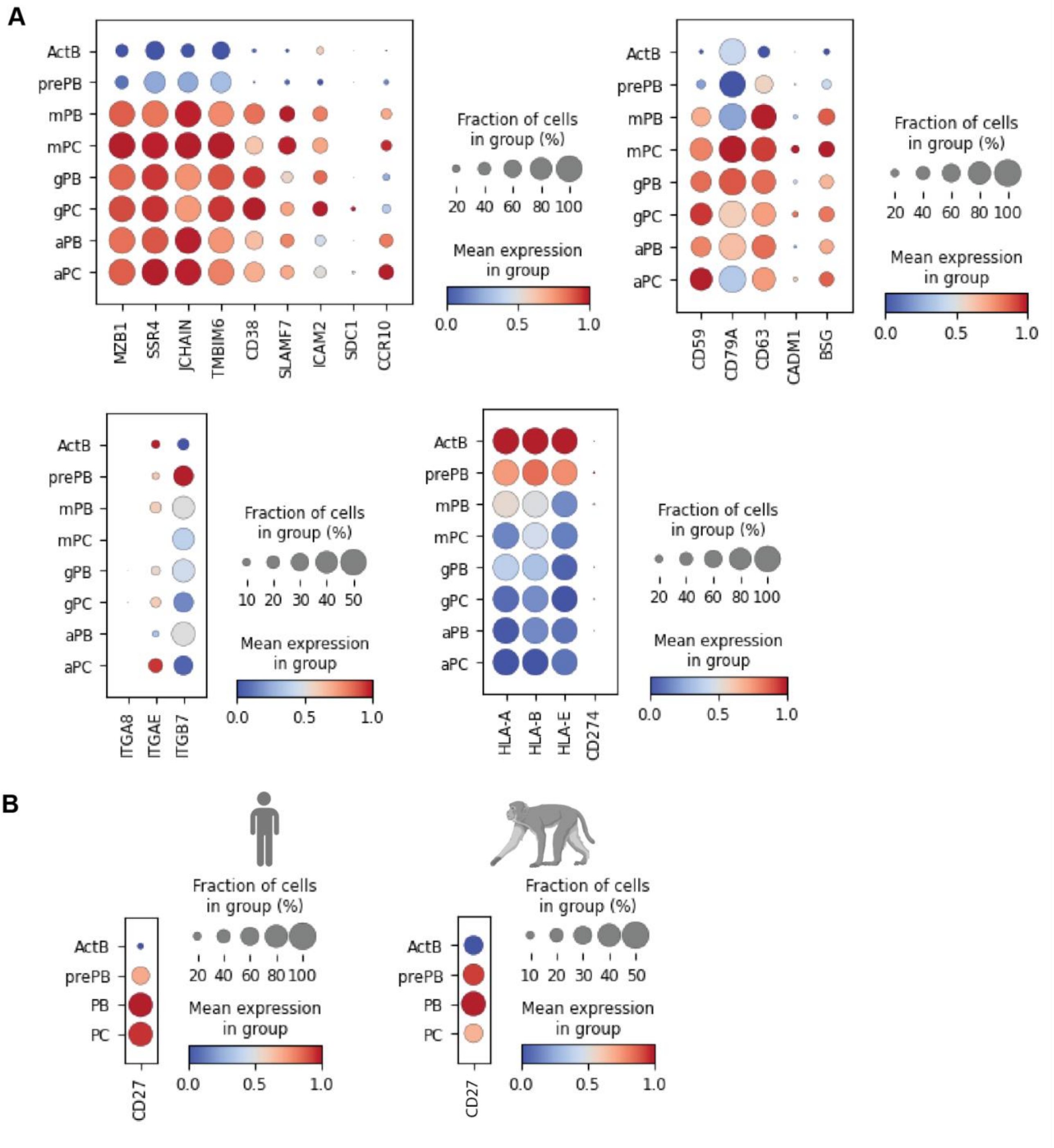

Figure S6. Comparison of human and monkey PC makers by scRNAseq analysis. Related to Figure 6. A. Dotplot visualization of *ex vivo* differentiated human PC: subsets with different isotypes are listed on y-axis and representative genes from Fig. 3D are listed along the x-axis. **B.** Dotplot visualization of *ex vivo* differentiated human and monkey PC: subsets are listed on y-axis and CD27 gene on x-axis.

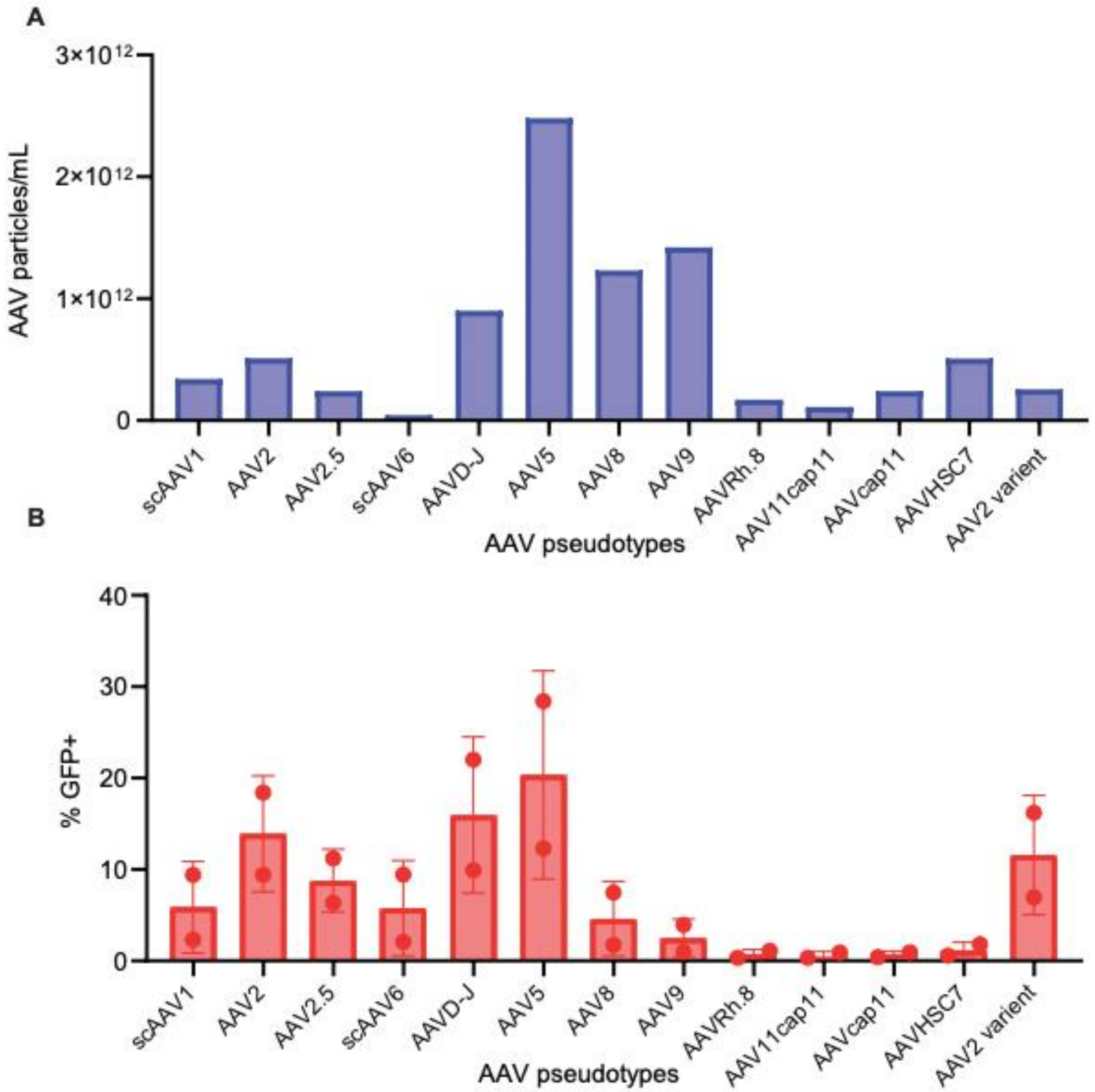

Figure S7. NHP B cell transduction with AAV. Related to Figure 5. A-B NHP B cells were transduced on day 3 of culture. 20% by volume was added of GFP encapsulated in different AAV pseudotypes, with varying titers **A.** and flow for GFP+ **B.** was run 2 days later.
