# Supplementary material for: Generation, expansion, gene delivery, and single-cell profiling in rhesus macaque plasma B cells
Source: Cell Rep Methods. 2024 Oct 14;4(10):100878. doi: 10.1016/j.crmeth.2024.100878 (PMC11573788; doi:10.1016/j.crmeth.2024.100878)
Supplement: Document S2. Article plus supplemental information [file mmc3.pdf]

Cell Reports
Methods

Article

Generation, expansion, gene delivery, and single-cell profiling in rhesus macaque plasma B cells

Graphical abstract

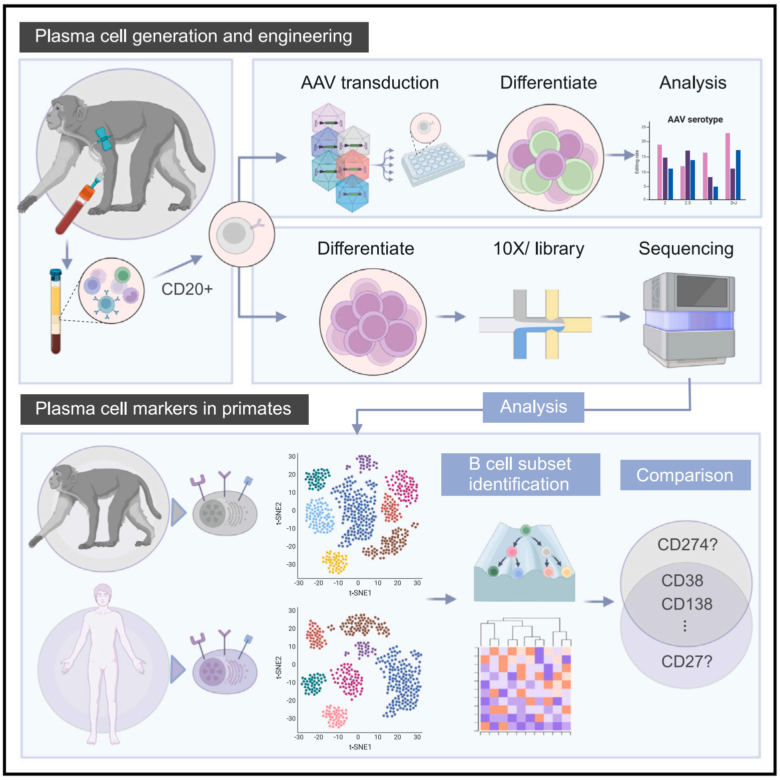

Highlights

- Expansion (10-fold) and differentiation (50% IgG) of NHP B cells into plasma cells
- Engineered rhesus monkeys using AAV(D-J) serotype achieve 60% transduction rate
- CD59 and CD79A can serve as surrogate markers for rhesus monkey plasma cells
- NHP plasma cells exhibit increases in PD-L1 and MHC class I expression

Authors

Rene Yu-Hong Cheng, Anna E. Helmers, Shannon Kreuser, ..., Christina Lopez, David J. Rawlings, Richard G. James

Correspondence

rickerj@u.washington.edu

In brief

Cheng et al. develop efficient methods for generating and engineering rhesus monkey plasma cells *ex vivo*. Using single-cell RNA sequencing, they identify both less-recognized plasma cell markers conserved in humans and unique markers specific to rhesus monkeys.

Yu-Hong Cheng et al., 2024, Cell Reports Methods *4*, 100878
October 21, 2024 © 2024 The Author(s). Published by Elsevier Inc.
https://doi.org/10.1016/j.crmeth.2024.100878

CellPress

Cell Reports Methods

Article

Generation, expansion, gene delivery, and single-cell profiling in rhesus macaque plasma B cells

Rene Yu-Hong Cheng,[1,2] Anna E. Helmers,[1] Shannon Kreuser,[1] Noelle Dahl,[1] Yuchi Honaker,[1] Christina Lopez,[1] David J. Rawlings,[1,3,4] and Richard G. James[1,2,5,6,7,*]
[1]Center of Immunotherapy and Immunity, Seattle Children Research Institute, Seattle, WA 98101, USA
[2]Molecular Engineering and Science Institute, University of Washington, Seattle, WA 98195, USA
[3]Department of Pediatrics, University of Washington, Seattle, WA 98195, USA
[4]Department of Immunology, University of Washington, Seattle, WA 98195, USA
[5]Department of Pharmacology, University of Washington, Seattle, WA 98195, USA
[6]Brotman-Baty Institute for Precision Medicine, Seattle, WA 98195, USA
[7]Lead contact
*Correspondence: rickerj@u.washington.edu
https://doi.org/10.1016/j.crmeth.2024.100878

MOTIVATION Protein and peptide drugs, including monoclonal antibodies, represent about 10% of the pharmaceutical market but face issues like poor solubility and short half-lives. Recent advances using engineered human plasma cells have shown promise in mice, but their safety and effectiveness need further testing in non-human primates, which provide better models for long-term evaluation.

SUMMARY

A key step in developing engineered B cells for therapeutic purposes is evaluation in immunocompetent, large-animal models. Therefore, we developed methods to purify, expand, and differentiate non-human primate (NHP; rhesus macaque) B cells. After 7 days in culture, B cells expanded 10-fold, differentiated into a plasma cell phenotype (CD38, CD138), and secreted immunoglobulin G. Using single-cell sequencing and flow cytometry, we verified the presence of plasma cell genes in differentiated NHP B cells and unearthed less-recognized markers, such as CD59 and CD79A. In contrast with human cells, we found that the immune checkpoint molecule *CD274* (PD-L1) and major histocompatibility complex (MHC) class I molecules were upregulated in NHP plasma cells in the transcriptional data. Lastly, we established the conditions for efficient transduction of NHP B cells with adeno-associated virus (AAV) vectors, achieving a delivery rate of approximately 60%. We envision that this work will accelerate proof-of-concept studies using engineered B cells in NHPs.

INTRODUCTION

Protein or peptide drugs, including monoclonal antibodies (mAbs), are a growing class of therapeutics that now constitute ~10% of the pharmaceutical market.[1] Although protein drugs have great promise due to their ability to specifically target pathways and cell types that have eluded small-molecule inhibitors, they have many drawbacks. These drawbacks can include poor solubility, requirement for mammalian or human post-translational modifications, and relatively short half-lives *in vivo*. Because of these drawbacks, protein drugs are relatively expensive and can be difficult to manufacture. Recently, we developed a cell-based method to deliver protein drugs, which we have successfully tested in immune-deficient mice. To do this, we generated *ex vivo* differentiated human plasma cells (PCs) and engineered them to produce protein drugs (including bispecific antibodies), and in immuno-deficient mice, we observed long-lasting antibody secretion for a year and potent tumor killing.[1–4]

We and others have previously shown that human[5] and murine[6] PCs can engraft in immune-deficient mice. However, the safety, feasibility, and scalability of engineered B cells have not been carefully evaluated in large-animal models. Larger animals, such as non-human primates (NHPs), more faithfully replicate human immune responses when compared to mice. They provide substantially larger tissues (e.g., bone marrow) for PC residency, more faithful routes of delivery, and the potential for increased longevity to evaluating the durability of therapy, all surpassing what is achievable in mice. The rhesus macaque animal model is increasingly used for the preclinical development of HIV vaccines, microbicides, and antiretroviral drugs.[7] While

This is an open access article under the CC BY license (http://creativecommons.org/licenses/by/4.0/).

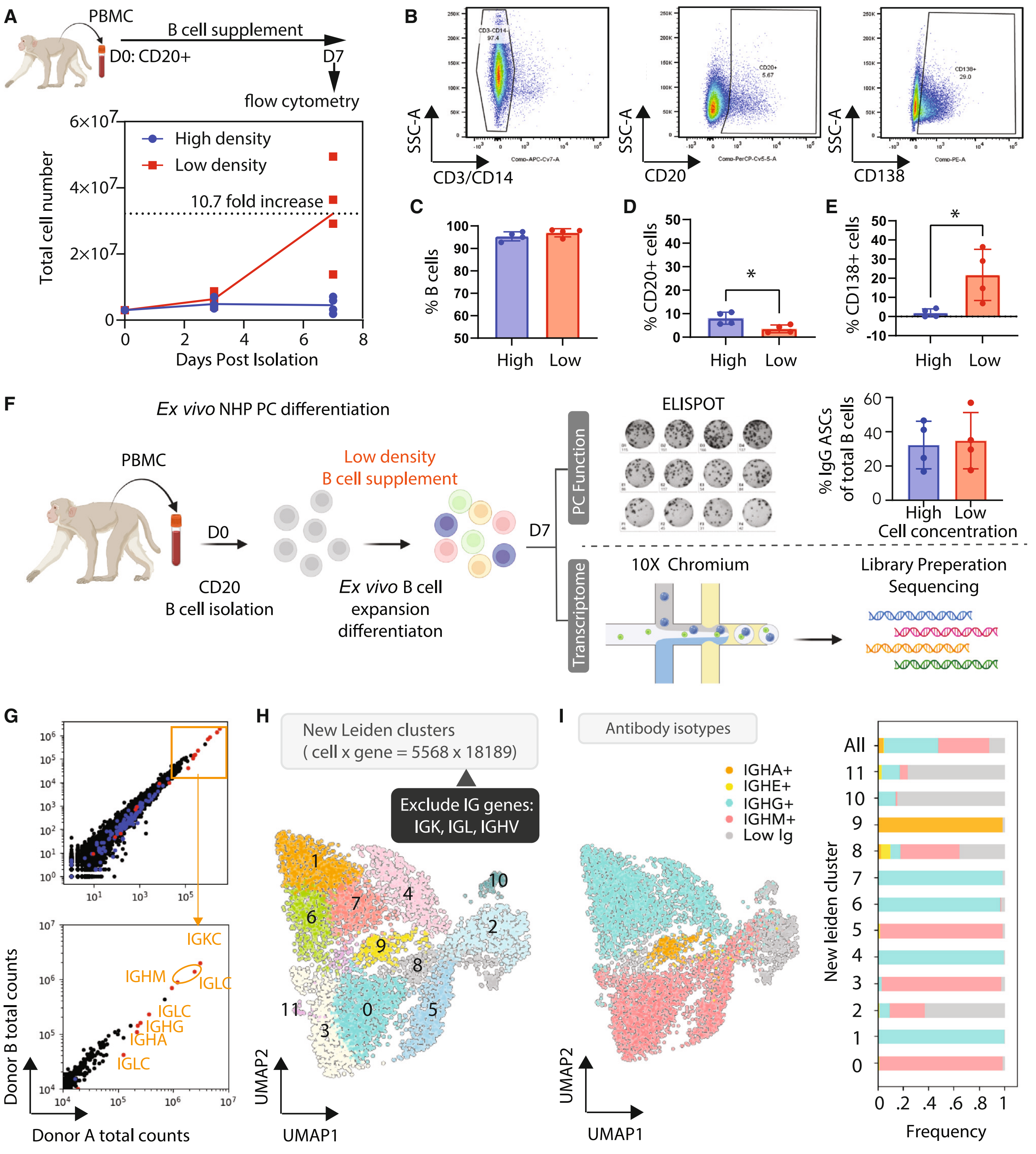

Figure 1. Monkey *ex-vivo*-differentiated PC transcriptome with high immunoglobulin expression

(A–E) To assess the impact of cell density, CD20$^+$ NHP B cells were cultured in a commercial B cell medium for 7 days (plated and maintained at 1.5 × 10^6 cells/mL versus plated at 2.5 × 10^5 cells/mL and maintained at 1 × 10^5 cells/mL; 4 donors, $n = 4$). At day 7 following isolation, the cells were analyzed using flow cytometry.

(A) Dynamic of total cell overtime.

(B) Representative flow cytometry plots of CD3/CD14, CD20, and CD138 expression.

(C) B cells (CD3$^-$CD14$^-$).

(D) Naive B cells (CD20$^+$).

(E) Plasma cells (CD138$^+$).

(legend continued on next page)

conditions for NHP B cell *in vitro* cultures[8] and suitability for adeno-associated virus (AAV) transduction[9] have been explored, a detailed molecular comparison of these cells with human PCs has not been achieved.

In vitro PC products derived from progenitor cells exhibit substantial heterogeneity.[1,5] Examining the genomic profile in NHP cell products is crucial for understanding how likely it is that experiments using these cells are going to mimic a human cell product. Using flow cytometry, Terstappen et al. showed that the canonical human PC marker CD38 is expressed by NHP bone marrow-derived PCs.[10] Furthermore, single-cell RNA sequencing (scRNA-seq) was used to demonstrate that NHP PCs from blood, bone marrow, and lymph nodes express similar factors to human primary PCs. They identified ICAM2 as a biomarker present in primary NHP PCs,[11] which they confirmed in humans. Comparisons between humans and NHPs can help provide a bridge between preclinical studies in NHPs and future clinical applications.

In this study, we refined the reagents and methods we previously developed[12] to establish a comprehensive workflow for expanding and engineering NHP PCs. This included the identification of a range of reagents for isolation, transduction with AAV, expansion, and differentiation of B cells sourced from peripheral rhesus macaque blood mononuclear cells. We anticipate that the level of B cell expansion in this system will enable multiple transfers of PCs at proportions relative to body weight, similar to those employed in previous murine studies of both human and murine PC longevity. Of particular significance, our work addresses a critical knowledge gap by providing molecular insights into the *ex vivo* differentiation of NHP PCs at the single-cell level. We demonstrated that NHP PC cultures exhibit remarkable heterogeneity and identified several potential markers that distinguish different stages of differentiation in NHP PCs. Additionally, through a comparative analysis of NHP single-cell data with previously generated human data, we observed a high degree of conservation in PC genes/markers (such as CD38, SLAMF7, ICAM2, MZB1, and CD59) across primates. However, in opposition to what is observed in human PCs,[1,13] we found that major histocompatibility complex (MHC) class I genes and CD274 (PD-L1) were significantly increased during the differentiation of NHP PCs, which could have implications for evaluating the immunogenicity of engineered NHP PCs. Finally, we validated several less-recognized PC markers using flow cytometry. Our findings confirmed that CD59 and CD79A can serve as surrogate markers for NHP PCs and that they are strongly correlated with canonical PC markers, including CD138.

RESULTS

Workflow for rhesus macaque *ex vivo* PC differentiation and illustration of the heterogeneity of the PC

Human cytokines and human-directed antibodies may not efficiently cross-react with NHP surface proteins. We empirically tested a suite of human reagents with rhesus macaque B cells. The first step in *ex vivo* B cell culture is the isolation of B cells from peripheral blood mononuclear cells. We identified an NHP CD20 positive selection kit that enabled the enrichment of NHP B cells at ~80% purity (flow cytometry panel in Table S1 and gating strategy in Figures S1 and S2A).

Next, we evaluated the efficacy of a human cytokine cocktail that included several T cell-dependent cytokines and CpG or a commercial human B cell expansion medium (Figure S2B). Following 7 or 13 days of culture, we found that, relative to defined cytokines, the commercial medium elicited greater B cell expansion and decreased non-B cell/T cell expansion (Figure S2C). To test the effect of seeding density on NHP B cell cultures, we cultured cells for 7 days in the commercial expansion cocktail at high (1.5 million cells mL^{-1}) and low (100,000 cells mL^{-1}) densities (Figures 1A–1E, S2D, and S2E). We found that NHP B cells grown at low density *ex vivo* exhibited significant increases in total numbers relative to those grown at high density (Figure 1A; ~10.5-fold compared to 1.5-fold). We found that B cells grown at low density also exhibited similar viability (Figure S2D) and purity (Figures 1B and 1C; ~95% as defined by the $CD14^{-}CD3^{-}$percentage). Upon evaluating the capacity for differentiation following culture, we found that the commercial media exhibited fewer $CD20^{+}$ (naive B) cells (Figures 1B and 1D; <10%), and higher percentages (up to 40%) of $CD138^{+}$ (PCs) cells (Figures 1B–1E). Finally, we also determined that ~50% of the cells produced immunoglobulin (Ig)G via ELISpot (top right, Figure 1F). These data indicated that NHP B cells expanded efficiently in commercial media and exhibited more rapid differentiation than we previously observed in human B cell conditions (13 days).[1,2]

The preliminary phenotype/ELISpot results suggest efficient *ex vivo* differentiation of NHP B cells into PCs. To more accurately assess heterogeneity in populations of differentiated NHP B cells, we expanded B cells from two donors at low density in commercial media for 7 days. At this point, we fixed the B cells and performed scRNA-seq on the mixed populations using the 10× Genomics platform (Figure 1F). Because the NHP genome was not fully annotated, especially within Ig and MHC genes, we made a workflow to annotate more genes associated with PC differentiation (Figure S3; Data S1).

To characterize NHP *ex-vivo*-differentiated PCs, we used unsupervised Leiden clustering and found the B cell populations to be heterogeneous, with 13 clusters in ~5,600 cells (Figure S4A). We categorized the cells by expression of the highly expressed (Figure 1G) Ig light-chain (LC) genes by drawing "gates" at the local minimum between the modes in each distribution (Figure S4B). Using this method, the clustering is primarily driven by the Ig genes, especially the LCs (IGKC and three IGLCs) (Figure S4B). To unearth additional differentiation-related clusters, we excluded the LCs and mapped LC-independent clusters (Figure 1H). As expected,

(C–E) Mean +/− SD is shown for barplots, and the statistical significance is determined by unpaired Student's t test. **p* value < 0.05.
(F) Schematic workflow of monkey *ex-vivo*-differentiated PC generation for functional (*n* = 4) and transcriptional (*n* = 2) profile characterization.
(G) Scatterplot of RNA total counts from two donors (*n* = 2). Red dots represent immunoglobulin constant genes, and blue dots represent immunoglobulin variable genes.
(H) UMAP Leiden clusters after removing immunoglobulin light-chain genes and variable genes.
(I) UMAP of antibody isotype. A bar graph of the percentage of antibody isotype in each cluster is shown.

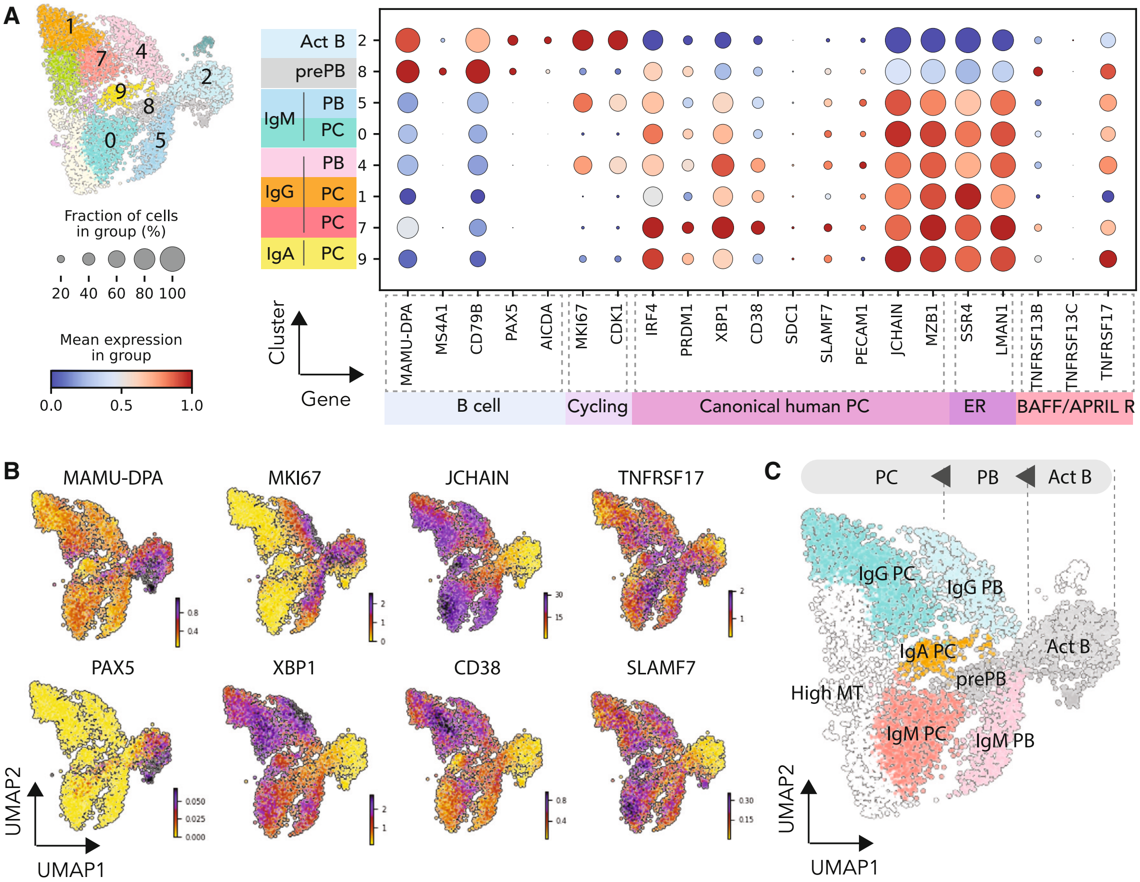

Figure 2. Monkey *ex-vivo*-differentiated PC subset classification and differential expression analysis for PCs

(A) Dot plot visualization of *ex-vivo*-differentiated PCs: subsets are listed on the y axis and genes (features) are listed along the x axis. Dot size represents the percentage of cells in a group expressing each gene; dot color indicates the normalized mean expression level in a group.

(B) Heatmap showing expression of representative genes of PC markers (*CD38*, *JCHAIN*, *XBP1*) and proliferating marker (*MKI67*).

(C) Cluster renamed to B cell subsets based on the gene features from (A) and (B).

after reclustering IGKC, IGLCx cells were distributed equally among the resulting 11 clusters (Figures 1H and S4C), which were now distributed into multiple subclusters for each antibody isotype (Figures 1H and 1I). Upon assessing unique molecular identifiers (UMIs) by cluster, we found that clusters 3 and 6 expressed higher mitochondrial genes and lower UMIs (Figure S4D). Additionally, clusters 10 and 11 had fewer than 100 cell counts and few detectable Igs (Figure 1I). Consequently, we excluded these clusters from further analysis (clusters 3, 6, 10, and 11). In summary, we predicted that the removal of the LC Igs and cleanup of non-B cell clusters would enable us to better understand the impact of lower expressed genes, such as transcription factors and surface marker genes.

Ex-vivo-differentiated rhesus macaque B cells form clusters analogous to those in human B cell cultures

To classify the cell types in the clusters, we looked at genes restricted to specific B cell clusters in human cells,[1,13] including activated B cells (*PAX5*, *MS4A1*, *CD79B*, and *MAMU-DPA*) and differentiated B cells (plasmablasts [PBs] and PCs; *IRF4*, *PRDM1*, *XBP1*, etc.) (Figures 2A and 2B). Cluster 2 exhibited low Ig counts and clear expression of activated B cell markers. Additionally, we categorized cluster 8 as pre-PBs (pre-PBs) due to the expression of activated B cell markers as well as increased *IRF4* and *PRDM1*. The remaining clusters exhibited similar expressions of human PC markers (Figures 2A and 2B), where the only obvious markers that qualitatively distinguished PBs from PCs were cell cycle genes (e.g., *MKI67* and *CDK1*). While some canonical markers, *XBP1* and *JCHAIN* (Figure 2B), were uniformly distributed among the NHP PB/PC populations, others like *SLAMF7*, *CD38*, and *TNFRSF17* (otherwise known as BCMA) were either poorly or differentially expressed within the NHP B cell subsets (Figures 2A and 2B). After assessment of human PB/PC markers and antibody isotypes, we were able to cluster *ex-vivo*-differentiated NHP B cells into activated B cells, pre-PBs, PBs, and PCs (Figure 2C).

CD59, CD274, and CD79A are robust rhesus macaque PC markers

To identify potential NHP PC markers, we used differential gene expression (DGE) analysis to find genes that are specifically expressed in PCs and/or PBs (Figure 3A). To accomplish this, we

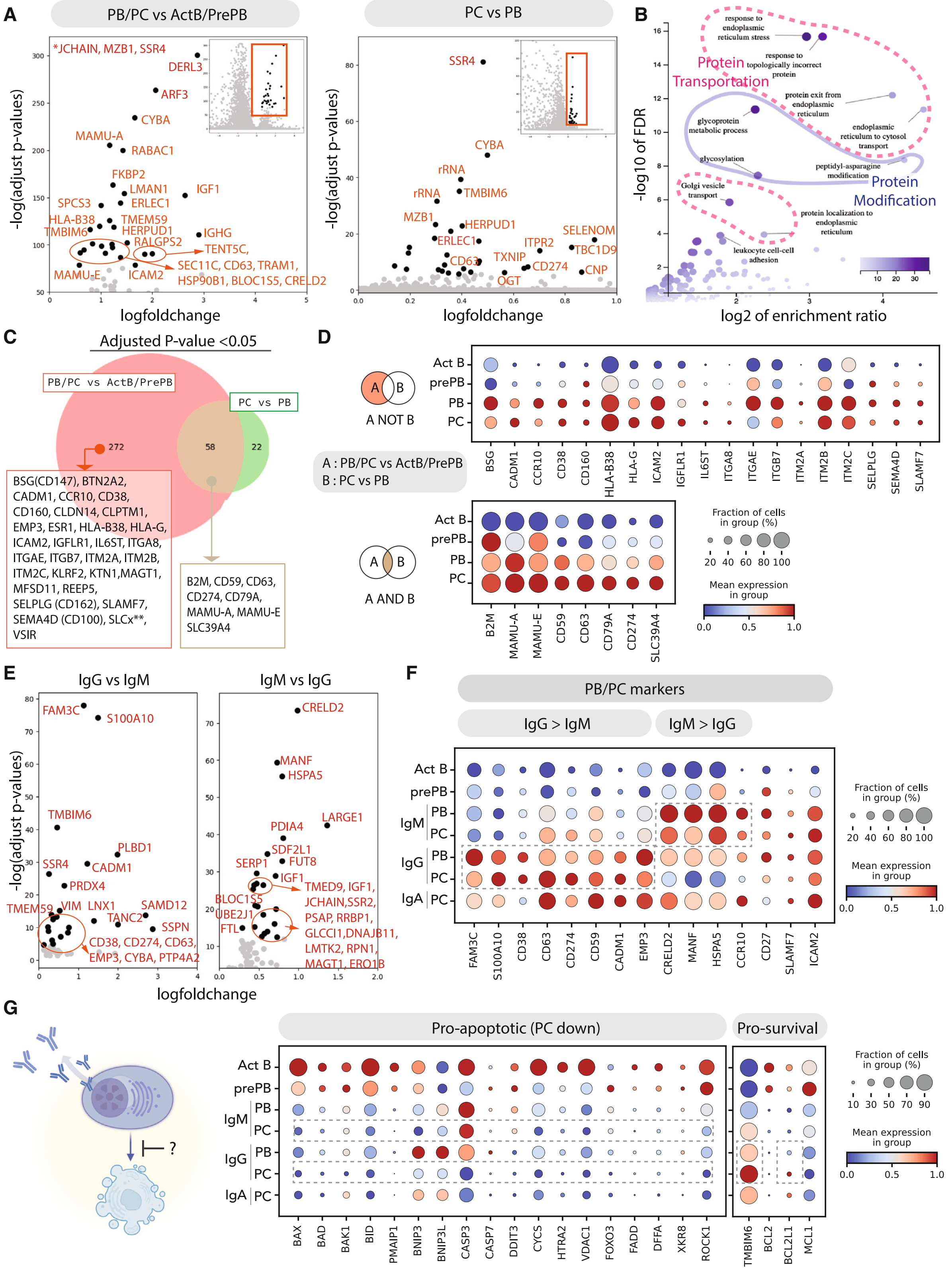

(legend on next page)

performed two comparisons: (1) clusters containing PBs/PCs versus undifferentiated B cells (activated B cells and pre-PBs), and (2) PCs versus PBs. Similar to previous observations in human cells,[1,2,13,14] we found that *SSR4*, *MZB1*, *CD63*, *JCHAIN*, *CYBA*, and *TMEM59* were enriched in PBs and PCs relative to undifferentiated B cells (Figure 3A).[1,2,13,14] Furthermore, the majority of genes upregulated in PBs/PCs were predominantly associated with the endoplasmic reticulum (ER), protein transportation, and protein modification (Figure 3B).

Because PBs/PCs exhibit a global program driven by activation of "master regulator" factors like IRF4, PRDM1, and XBP1,[15,16] we were not surprised to find pronounced differences between PBs/PCs and undifferentiated B cells relative to the differences between PCs and PBs (Venn diagram showing surface markers, Figure 3C). Several genes enriched in PBs/PCs are expressed at similar levels in both cell subsets (Figure 3D, top), such as canonical human PC markers, *CD38* and *SLAMF7*, and *ICAM2*, which was previously reported to mark NHP PCs (CD102) (Figure 3D).[11] We plotted the expression of the canonical PC marker SDC1 (CD138; Figure S4E) because of its low detection in scRNA-seq data. In contrast, a subset of surface markers are upregulated in PBs/PCs and are increased in PCs relative to PBs (Figure 3C, bottom; e.g., *CD59*, *CD274*, *CD79A*, *MAMU-A*, and *MAMU-E*). In contrast to previous findings in human cells,[13] we observed an upregulation of several MHC class I molecules in NHP PBs/PCs. We previously reported that CD59 is highly expressed in human IgG PCs and correlated with Ig secretion.[2] Because of its robust expression in NHP PCs and its correlation with PC function in human cells, we propose that CD59 and possibly other genes in this dataset are likely to be useful surrogate markers for functional NHP PCs.

To understand whether the identity of the antibody isotype correlated with transcriptional differences, we performed a DGE analysis of IgM and IgG PBs/PCs (Figures 3E and 3F). We found that some canonical PC markers, including *CD38*, and some non-canonical markers were expressed at higher levels in IgG relative to IgM PBs/PCs. In contrast, genes associated with ER quality control were enriched in IgM (*CRELD2*, *MANF*, and *HSPA5*) PBs/PCs. It is possible that these differentially expressed markers relate to the unique functions of IgM and IgG PCs in short- and long-term humoral immunity.

Finally, given the differential expression of apoptotic pathways in long-lived human PCs,[13,17,18] we investigated the presence of apoptotic genes in NHP *ex-vivo*-differentiated PCs. Similar to the description in human cells, we found that pro-apoptotic genes are generally downregulated during NHP PC differentiation (Figure 3G), with the exception of *BNIP3*, *BNIP3L*, and *CASP3*, which were upregulated in PBs and then downregulated in PCs. Additionally, among pro-survival (anti-apoptotic) genes, we observed the upregulation of *TMBIM6* (one of the highest IgG DGE genes in Figure 3E) and *BCL2L1* in IgG PCs (Figure 3G). Collectively, these scRNA-seq data demonstrate that *ex-vivo*-derived NHP B cells produced a heterogeneous population that included subsets of PCs with similar transcriptional profiles to long-lived human PCs.

Dynamic expression and kinetic differential analysis of NHP potential PC markers

mRNA splicing can provide supportive information about the progression of cellular differentiation. Upon upregulation of gene expression, unspliced transcripts are often present at a relatively high proportion relative to that observed in steady-state conditions.[19] RNA velocity, or the relative ratio of unspliced to spliced RNA, can be used to infer the upregulation of transcripts in cell subsets within scRNA datasets.[19] As expected, the velocities of IgG, IgM, and IgA were increased in PBs/PCs expressing each isotype, although there appeared to be some heterogeneity within each group (top, Figure 4A).

In support of the steady-state mRNA data, we found that *CD274*, *CD59*, and *MAMU-A* exhibited positive RNA velocity in IgG^{+} PC clusters (Figure 4A). In contrast, genes enriched in activated B cells (e.g., *MAMU-DPA* and *PAX5*) only exhibited positive RNA velocity in non-PC clusters (Figure S5A). Next, we used scVelo, an unsupervised method that leverages RNA velocity to infer the progression of cells along a developmental trajectory, or pseudotime. The pseudotime trajectory generated by the model proceeded from activated cells (right side of the uniform manifold approximation and projection [UMAP], Figure S5B) to IgG PCs (left side of the UMAP, Figure S4B). Using the partition-based graph extraction analysis (PAGA) connection analysis,[20] we calculated the transition probability from one cluster to another (Figure 4B, bottom). The trajectory analysis of activated B cells into antibody-secreting PCs in the NHP system was similar to that observed in human cells.[21]

After ordering cells based upon velocity pseudotime, we can see the progression/dynamic of genes of interest along the PC differentiation (Figures 4C and S5C). Similar to the connection analysis, the gene expression trajectories followed canonical B cell

Figure 3. Monkey PCs' potential markers from differentially expressed genes

(A) Volcano plot of differential expression genes: PBs/PCs versus activated B cells/pre-PBs and PCs versus PBs. Only the top 30 differential genes are highlighted as black dots. **JCHAIN*, *MZB1*, and *SSR4* are infinitely small adjusted *p* values and not plotted in the plot.

(B) Volcano plot of enriched gene set from an over-representation analysis using the single-cell anslysis in Python algorithm. Heatmap represents the number of genes enriched in each gene set.

(C) Venn diagram of genes upregulated in PB/PCs and PCs by comparison with activated B cells/pre-PBs and PBs with adjusted p value <0.05. Only surface markers for some transmembrane genes are highlighted.

(D) Dot plot visualization of *ex-vivo*-differentiated PCs: subsets are listed on the y axis and genes (features) are listed along the x axis.

(E) Volcano plot of differential expression genes: IgG PBs/PCs versus IgM PBs/PCs (left) and IgM PBs/PCs versus IgG PBs/PCs (right). Only the top 25 differential genes are highlighted as black dots.

(F) Dot plot visualization of *ex-vivo*-differentiated PBs/PCs: subsets with different isotypes are listed on the y axis and representative surface marker genes or genes from (E) are listed along the x axis.

(G) Schematic cartoon of a PC's anti-apoptotic program. Dot plot visualization of *ex-vivo*-differentiated PBs/PCs: subsets with different isotypes are listed on the y axis and representative anti-/pro-apoptotic genes are listed along the x axis.

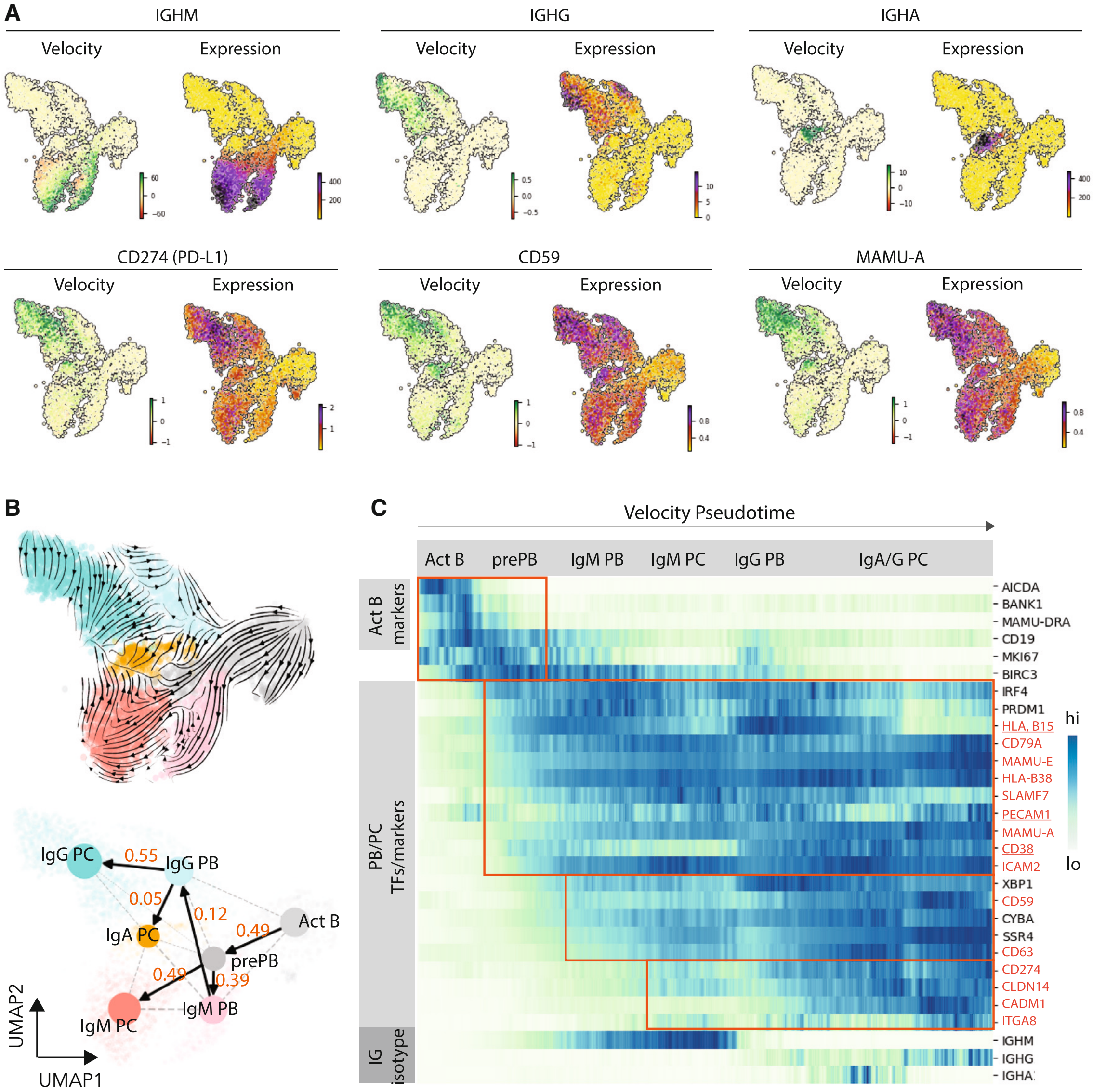

Figure 4. Monkey PCs' dynamic expression

(A) Heatmap of indicated gene velocity and RNA expression level superimposed onto UMAP.

(B) Velocities derived from the stochastic model and visualized as streamlines in a UMAP-based embedding (top). PAGA/transition confidence probability from one node to the next node (bottom).

(C) Expression heatmap of genes shows gene regulation along plasma cell development and genes upregulated in PCs from differentially expressed gene (DEG) analysis. The velocity pseudotime is shown along the x axis and the indicated genes are shown on the y axis. Potential PC markers are highlighted orange.

differentiation, starting from cells with markers of B cell activation (*AICDA*, MHC class II, *CD19*, and *BIRC3*), followed by the upregulation of transcription factors associated with PB/PC differentiation (*IRF4*, *PRDM1*, etc.). Potential PC markers can be classified into three groups based on the timing of the onset: (1) genes initially expressed in pre-PBs (*CD79A*, *MAMU-E*, *HLA-B38*, *MAMU-A*, and *ICAM2*), (2) genes initially expressed in pre-PBs/PBs (*CD59* and *CD63*), and (3) genes initially expressed in PCs (*CD274*, *CLDN14*, *ITGA8*, and *CADM1*). Some genes did not follow the patterns above. *HLA-B15* was upregulated in PBs but downregulated in PCs. *CD38* was expressed in pre-PBs but only upregulated in IgG PBs/PCs. *PECAM1* (encoded *CD31*) is isotype specific: it was more abundant in IgG PBs/PCs relative to either IgM or IgA PBs/PCs. By applying kinetic differential analysis (Figure S5D), *CD274* was also identified as being differentially upregulated in IgG PCs, validating its importance as a potential marker for NHP PCs.

Flow cytometry validated that CD59 and CD79A can serve as surrogate PC markers

In order to determine if markers identified by RNA sequencing could be used to phenotype PCs by flow cytometry, B cells

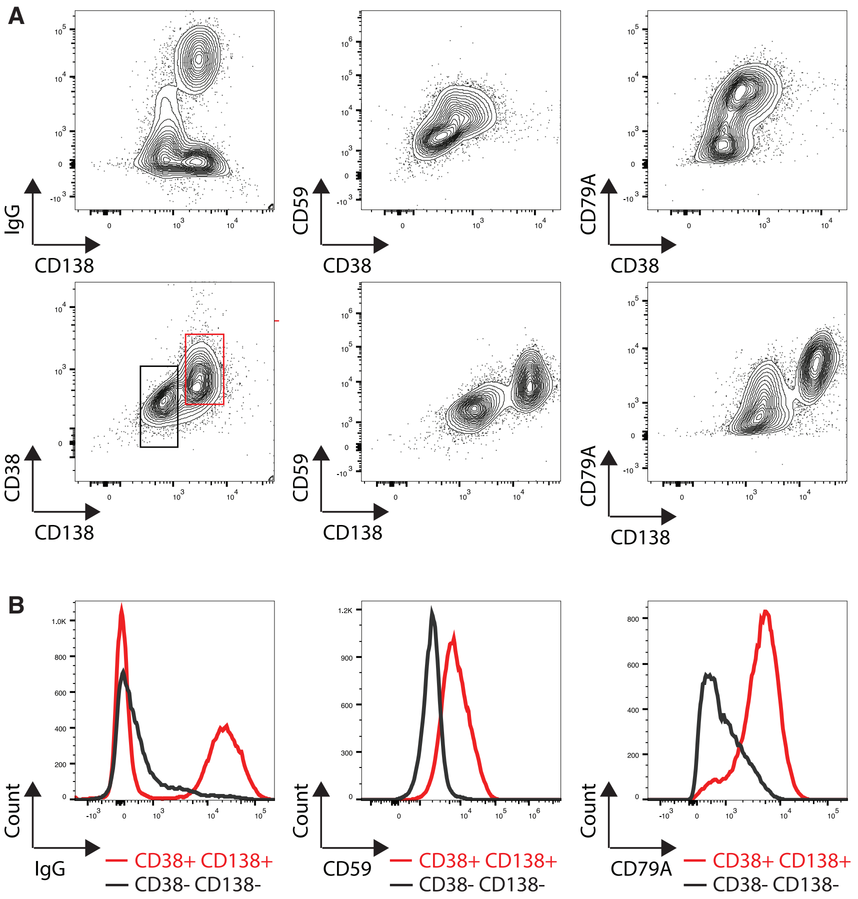

Figure 5. Using flow cytometry to validate non-canonical markers for NHP PCs

(A) Flow plot of canonical PC markers (CD38, CD138) and IgG versus non-canonical markers.

(B) IgG, CD59, and CD79A expression in $CD38^{-}$ $CD138^{-}$ B cells versus $CD38^{+}CD138^{+}$ PCs. CD59 and CD38 were stained prior to fixation, while CD138, CD79A, and IgG were stained after fixation.

were isolated and cultured at low density in commercial media for 8 days prior to analysis by flow cytometry.

Cells were stained for canonical PC markers (CD38, CD138, and IgG) as well as non-canonical PC markers (CD59 and CD79A). CD59 and CD79A were more highly expressed in $CD138^{+}$ cells, a pattern that was less clear in $CD38^{+}$ cells (Figure 5). Furthermore, we found that IgG, CD59, and CD79A were expressed at higher abundance in B cells expressing canonical PC markers ($CD38^{+}CD138^{+}$) relative to B cells that did not ($CD38^{-}CD138^{-}$; Figure 5B). Based on these results, we concluded that CD59 and CD79A are likely surrogate markers for NHP PCs.

Primate: Rhesus macaque PCs in comparison with human PCs

Previously, we used CITE-seq to characterize the *ex-vivo*-differentiated human PCs with CD38, CD138, and IgM oligo-conjugated antibodies.[1] We used these markers to categorize human B cells into subsets based on surface expression: activated B cells ($IgM^{hi}CD38^{-}CD138^{-}$), pre-PBs ($IgM^{hi}CD38^{lo}CD138^{-}$), PBs ($IgM^{lo}CD38^{+}CD138^{-}$), and PCs ($IgM^{lo}CD38^{+}CD138^{+}$) (Figure 6A, left UMAP), which we validated via quantification of the B cell and PC transcription factors *PAX5* and *XBP1*, respectively (Figure 6B). In PCs, the majority of antibody isotype is IgG (Figure 6A, right UMAP). *Ex-vivo*-differentiated NHP PCs primarily expressed IgG but exhibited higher proportions of IgM than we observed in human cultures. Additionally, the NHP PCs exhibited similar maturity (e.g., PC markers in Figure 3, ER/Golgi features in Figure 3B, and ELISpot in Figure 1F) following 7 days of culture to what humans achieved in 13 days.

We proceeded to compare the NHP PC markers we identified (Figure 3) with those in human PCs. As anticipated, canonical markers such as *CD38* and *SLAMF7* exhibited higher expression in PB/PC stages. In both species, we found that *CD38* exhibited higher expression in IgG PCs (Figures 6C and S6A), and the paramount PC marker CD138 (*SDC1*) was expressed at higher levels in PCs but exhibited low RNA counts in all subsets (Figures 2A and 6C). *CCR10*, canonically expressed in mucosal IgA PCs, was detected specifically in IgA PCs in both species (Figures 6C and S6A). Finally, we found several understudied markers, including *CD59*, *CD79A*, *CD63*, *CADM1*, and *BSG* (CD147), to be upregulated in both human and NHP PCs, suggesting further research is justified to clarify potential roles for these genes in PC biology. CD27 is highly expressed in human PBs and PCs. In NHPs, CD27 is only upregulated in pre-PBs and PBs, and its expression is significantly lower in IgG and IgA PCs (Figure 3F). It has been reported previously that CD27 is not a critical marker for NHP PCs (Figure S6B).[22,23] Of particular interest was the behavior of MHC class I genes. *HLA-A* and *HLA-E* (which correspond to *Mamu-A* and *Mamu-E*), while highly expressed in all human B cell subsets, are downregulated in PCs (Figure 6C). This result directly contrasts with our observations in NHP PCs (Figure 3D) whereby the MHC class I genes are upregulated during PC maturation. Additionally, *CD274* (PD-L1), which is dramatically upregulated in NHP PCs, was hardly detectable in human pre-PBs and PCs (Figure 6C, right). Collectively, these data imply that while the transcriptional signatures of *ex-vivo*-derived PCs are well conserved between NHPs and humans (Figure 6D), there may be substantive differences in the expression of T cell regulators (MHC class I and PD-L1) between the species.

Efficient transduction of NHP B cells with the AAV serotypes

To assess the potential for transduction of NHP B cells with recombinant AAV vectors, we isolated B cells, cultured them

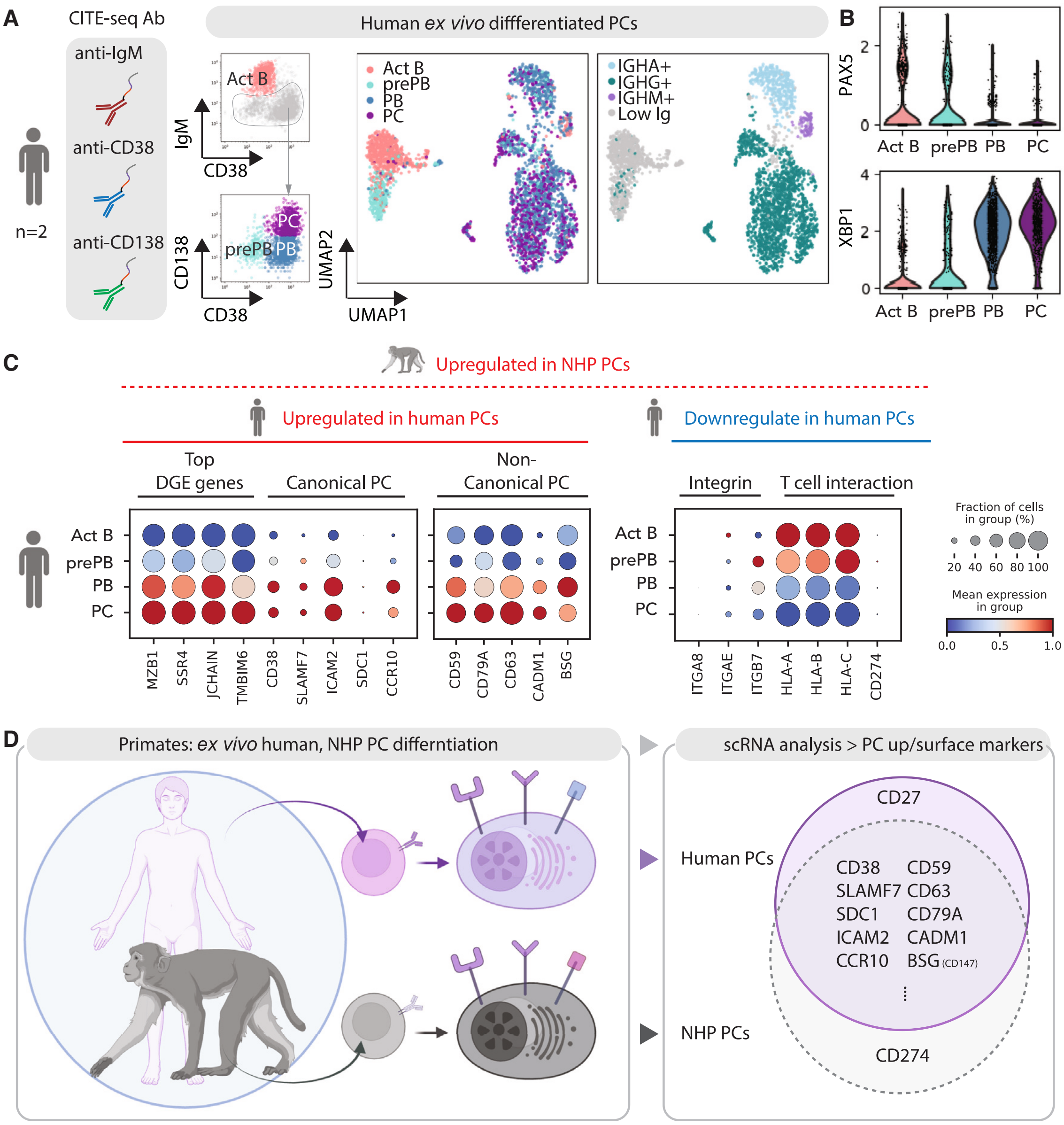

Figure 6. Comparison of human and monkey PC makers by scRNA-seq analysis

(A) *Ex-vivo*-differentiated human PCs with CITE-seq. Classification of B cell subsets categorized by the indicated protein markers: IgM^{hi}CD38^{lo} (activated B cells, ActB), IgM^{lo}CD38^{lo} (pre-PBs), CD38^{hi}CD138^{lo} (PBs), and CD38^{hi}CD138^{hi} (PCs). B cell subsets and isotype are superimposed onto UMAP; day 13 B cells (n = 2,897) are from two biological replicates.

(B) Violin plot of indicated genes.

(C) Dot plot visualization of *ex-vivo*-differentiated human PC: subsets are listed on the y axis and representative genes from Figure 3D are listed along the x axis.

(D) Schematic cartoon summary of human PC markers compared with monkey PC markers from scRNA analysis. *MHC class I is expressed in most cell types but is not ideal as a marker for NHP PCs.

briefly, and exposed the cells to a panel of self-complementary AAV vectors. We assessed a series of AAVs with alternative serotypes, each carrying a GFP expression cassette to permit efficient tracking of transduced cell populations. Following exposure to AAV, B cells were maintained in culture for 9 more days prior to assessment of GFP positivity in CD3^{-}CD14^{-} cells. In a volume-matched experiment (20% AAV by volume), we found that AAV D-J serotypes exhibited the most efficient transduction (Figures S7A and S7B; Table S2). To rule out the impact of titer, we directly compared transduction with the 2.5 (control) and D-J serotypes using the same titers (Figure 7A). We found that 2.5 and D-J exhibited similar transduction efficiencies at day 2, but D-J increased the efficiencies to 60% at day 4 and retained high percentages at late time points (day 10; Figure 7B). Next, to determine whether AAV D-J is useful for the delivery of a candidate,

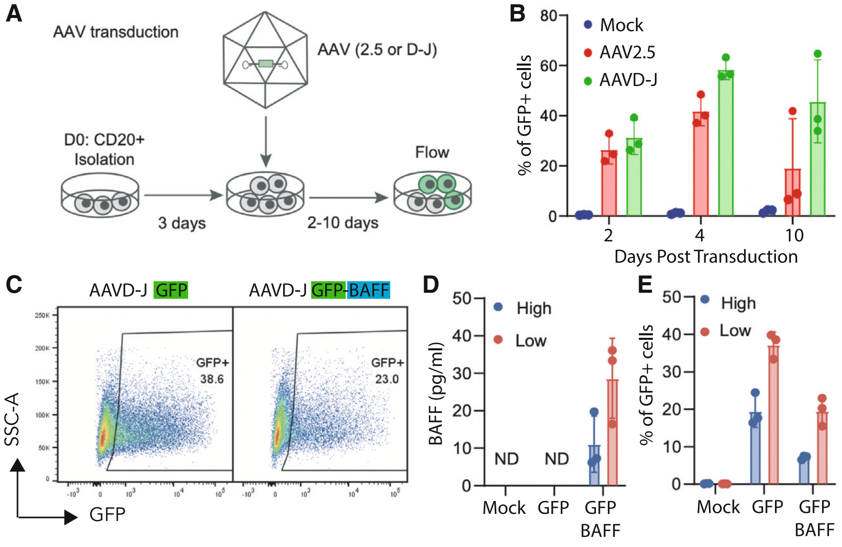

Figure 7. Efficient transduction of NHP B cells with the AAV

(A) Diagram of the AAV and transduction and culturing protocol.

(B) NHP CD20$^+$ B cells were cultured for 3 days before transduction with the indicated AAV pseudotypes expressing GFP (MOI = 4×10^4). Transduction efficiency was quantified by flow cytometry at the indicated time points.

(C–E) NHP CD20$^+$ B cells grown at high (1.5×10^6 cells/mL) or low (2.5×10^5 cells/mL) density were transduced with either AAV D-J GFP or AAV D-J GFP-BAFF (3 donors, $n = 3$).

(C) Representative flow plot of GFP and GFP-BAFF transduced cells.

(D) Bar plot of BAFF secretion was quantified 4 days post-transduction.

(E) Bar plot of percentage of GFP$^+$ cells with indicated high or low cell density and AAV.

(B), (D), and (E) Mean +/− SD is shown for barplots.

physiologically relevant payload in NHP B cells, we transduced NHP B cells with single-stranded AAV expressing the cytokine B cell activating factor (BAFF) *cis*-linked to GFP. While the transduction rates using the BAFF AAV vector were slightly lower than the self-complementary GFP vector (Figure 7C), we detected high levels of BAFF secretion (Figure 7D) in NHP B cells. We also tested whether transduction was different in B cells cultured at different densities. We found that NHP B cells cultured at low density were transduced at higher rates than those cultured at high densities (Figure 7E), and produced more BAFF in culture (Figure 7D). In conclusion, because AAV D-J generally produces higher titers, we predict that the DJ serotype will be more useful than 2.5 for the transduction of NHP B cells.

DISCUSSION

An important proof-of-concept experiment for developing a future human PC-based therapy is the assessment of PC survival and production capacity in an immunocompetent system. Although NHP models have been used extensively to study the response to vaccines[24] and PC longevity,[25] we are unaware of comprehensive studies optimizing the expansion/differentiation of NHP PCs, describing their heterogeneity, or comparing them to human PCs. To address this challenge, we developed methods to purify, expand, and differentiate rhesus macaque B cells *ex vivo.* We achieved a 10-fold expansion of NHP B cells and observed efficient differentiation into antibody secretion cells (~90% Ig^{high} cells). Using scRNA-seq, we confirmed that after 7 days in culture, the majority of differentiated NHP B cells express markers associated with antibody-secreting cells, including transcription factors, genes regulating protein secretion, and Ig molecules. Furthermore, we identified several surrogate NHP PC markers (CD59, CD79A) with flow cytometry and compared the transcriptional profiles of NHP and human *ex-vivo*-derived PCs. Finally, we identified the serotypes (D-J) and conditions necessary for the efficient transduction of NHP B cells with AAV vectors, which we hope will be used in homology-directed repair studies to generate cells producing biologic drugs.

Of particular significance, our observations revealed an upregulation of several MHC class I molecules in NHP (*B2M*, *HLA-A*, *HLA-E*, *HLA-G*) PBs/PCs. While it is well established that MHC class II molecules are downregulated in human PCs, the expression of MHC class I genes by PCs in other species has received relatively limited characterization.[26–28] As shown in our human PC scRNA-seq data (Figure 6) and data generated by other groups,[13] MHC class I genes are downregulated in human PCs. We also observed the upregulation of PD-L1 (*CD274*) in NHP, but not human, PCs.[1] PD-L1 is a cell surface protein expressed by various cell types, including cancer cells. PD-L1 expression by cancer inhibits cytotoxic T cell function and promotes immune evasion.[29–31] Because MHC class I and PD-L1 are likely to drive opposing effects on T cell function, it will be important to understand whether these differences between NHP and human PCs have functional impacts on the presentation of foreign antigens by NHP PCs and the impact on T cell surveillance. It is possible that the upregulations of MHC class I and PD-L1 are linked and play a functional role to maintain homeostasis and prevent excessive killing of PCs. However, this is an area that warrants additional evaluation.

To address the question how many PCs will be required in primates to generate a neutralizing dose of antibody or relevant levels of alternative therapeutic proteins, there are several variables to be considered: the half-life of the protein, the effective dose, the production of protein per PC, and the engraftment capacity of the PC product. Fortunately, for most clinical mAbs, we know the half-life and the required neutralizing dose. We also know fully differentiated long-lived PCs are estimated to produce ~50 pg/cell/day,[32] and even in an immuno-deficient mice setting with limited humanized factors (interleukin [IL]-6 and BAFF), we can still observe at least 50 μg/mL IgG from a one-time 10 million PC infusion after 150 days.[1] We can expect that with an immunocompetent primate and the larger capacity for PCs, it is likely to

achieve more than 50 μg/mL therapeutic antibodies, which is an effective dose to neutralize certain viruses.[33] It is important to note that this topic requires further study, but it is beyond the scope of our current research.

Upon successful development of engineered primate PCs, we expect that production of a non-clinical cell therapy product could be multiplexed and delivered singly or repeatedly into autologous donor recipients. In summary, these results suggest that proof-of-concept experiments using one or more doses of engineered primate PCs are likely to be feasible with minor adjustments to the protocol presented here.

Limitations of the study

The NHP donors were randomized at the time of purchase from a commercial source; we do not have access to the historical health information for these donors. While our data show no discrepancies among the donors, we are unable to perform a careful study that accounted for heterogeneity among them. This limitation should be considered when interpreting the results of our study. While *ex-vivo*-generated rhesus monkey PCs resemble human PCs in function (as shown by ELISpot) and transcriptomic features, it is uncertain whether the markers we identified will be sustained long term *in vivo*. Further studies are needed to access the timing and functional relevance of these makers, as well as the longevity of these *ex-vivo*-generated PCs or engineered PCs *in vivo*.

RESOURCE AVAILABILITY

Lead contact

Further information and requests for resources and reagents should be directed to and will be fulfilled by the lead contact, Richard G. James (rickerj@u.washington.edu).

Materials availability

This study did not generate new reagents. Commercially available reagents are listed in the key resources table.

Data and code availability

- The scRNA-seq data are available at GEO: GSE247023.
- Code is available here: https://github.com/Rene2718/Rhesus-Macaque-Plasma-B-Cells-single-cell-RNAseq-. An archival DOI is listed in the key resources table.
- Any additional information required to reanalyze the data reported in this work paper is available from the lead contact upon request.

ACKNOWLEDGMENTS

We would like to thank other members of the James lab for helpful comments and discussion in the preparation for this manuscript. We would like to thank the viral core at the Center for Immunity and Immunotherapies at Seattle Children's Research Institute for reagents. This research was supported by the Bill and Melinda Gates Foundation (INV-002355). This work was supported in part by the Seattle Children's Research Institute (SCRI) Program for Cell and Gene Therapy (PCGT), the Children's Guild Association Endowed Chair in Pediatric Immunology, and the Hansen Investigator in Pediatric Innovation Endowment (to D.J.R.).

AUTHOR CONTRIBUTIONS

R.Y.-H.C., S.K., Y.H., D.J.R., and R.G.J. designed the study. R.Y.-H.C., A.E.H., S.K., and R.G.J. wrote the manuscript with advice from D.J.R. S.K., N.D., A.E.H., and C.L. established the culture system and performed AAV editing and phenotyping. S.K. and R.Y.-H.C. ran the 10× experiment, prepared libraries, and ran the sequencing. R.Y.-H.C. analyzed the 10× scRNA-seq data. A.E.H. performed and analyzed the flow cytometry to validate marker expression in NHP cells.

DECLARATION OF INTERESTS

R.G.J. and D.J.R. hold equity in and serve on the scientific advisory board of Be Biopharma, Inc. S.K. is currently an employee at Astellas Pharma, Y.H. is currently an employee of Sonoma Biotherapeutics, and C.L. is currently an employee of Sartorius AG.

STAR★METHODS

Detailed methods are provided in the online version of this paper and include the following:

- KEY RESOURCES TABLE
- METHOD DETAILS
  - B cell isolation
  - B cell culture conditions
  - Flow cytometry
  - ELISpot and ELISAS
  - Virus production and transduction
  - Single cell RNA sequencing
  - Single-cell RNA-seq analysis
  - Comparison between monkey and human
- QUANTIFICATION AND STATISTICAL ANALYSIS

SUPPLEMENTAL INFORMATION

Supplemental information can be found online at https://doi.org/10.1016/j.crmeth.2024.100878.

Received: January 17, 2024
Revised: July 18, 2024
Accepted: September 20, 2024
Published: October 14, 2024

REFERENCES

1. Cheng, R.Y.-H., Hung, K.L., Zhang, T., Stoffers, C.M., Ott, A.R., Suchland, E.R., Camp, N.D., Khan, I.F., Singh, S., Yang, Y.-J., et al. (2022). Ex vivo engineered human plasma cells exhibit robust protein secretion and long-term engraftment in vivo. Nat. Commun. *13*, 6110.
2. Cheng, R.Y.-H., de Rutte, J., Ito, C.E.K., Ott, A.R., Bosler, L., Kuo, W.-Y., Liang, J., Hall, B.E., Rawlings, D.J., Di Carlo, D., and James, R.G. (2023). SEC-seq: association of molecular signatures with antibody secretion in thousands of single human plasma cells. Nat. Commun. *14*, 3567.
3. Vamva, E., Ozog, S., Leaman, D.P., Yu-Hong Cheng, R., Irons, N.J., Ott, A., Stoffers, C., Khan, I., Goebrecht, G.K.E., Gardner, M.R., et al. (2023). A lentiviral vector B cell gene therapy platform for the delivery of the anti-HIV-1 eCD4-Ig-knob-in-hole-reversed immunoadhesin. Mol. Ther. Methods Clin. Dev. *28*, 366–384.
4. Hill, T.F., Narvekar, P., Asher, G.D., Edelstein, J.N., Camp, N.D., Grimm, A., Thomas, K.R., Leiken, M.D., Molloy, K.M., Cook, P.J., et al. (2024). Human plasma cells engineered to secrete bispecifics drive effective in vivo leukemia killing. Mol. Ther. *32*, 2676–2691.
5. Hung, K.L., Meitlis, I., Hale, M., Chen, C.-Y., Singh, S., Jackson, S.W., Miao, C.H., Khan, I.F., Rawlings, D.J., and James, R.G. (2018). Engineering Protein-Secreting Plasma Cells by Homology-Directed Repair in Primary Human B Cells. Mol. Ther. *26*, 456–467.
6. Moffett, H.F., Harms, C.K., Fitzpatrick, K.S., Tooley, M.R., Boonyaratanakornkit, J., and Taylor, J.J. (2019). B cells engineered to express pathogen-specific antibodies protect against infection. Sci. Immunol. *4*, eaax0644. https://doi.org/10.1126/sciimmunol.aax0644.

7. Van Rompay, K.K.A. (2012). The use of nonhuman primate models of HIV infection for the evaluation of antiviral strategies. AIDS Res. Hum. Retroviruses *28*, 16–35.
8. DeLaura, I., Schroder, P.M., Yoon, J., Ladowski, J., Anwar, I.J., Ezekian, B., Schmitz, R., Fitch, Z.W., Kwun, J., and Knechtle, S.J. (2022). A novel method for in vitro culture and expansion of nonhuman primate B cells. J. Immunol. Methods *511*, 113363.
9. Hartweger, H., Gautam, R., Nishimura, Y., Schmidt, F., Yao, K.-H., Escolano, A., Jankovic, M., Martin, M.A., and Nussenzweig, M.C. (2023). Gene Editing of Primary Rhesus Macaque B Cells. J. Vis. Exp. https://doi.org/10.3791/64858.
10. Terstappen, L.W., Johnsen, S., Segers-Nolten, I.M., and Loken, M.R. (1990). Identification and characterization of plasma cells in normal human bone marrow by high-resolution flow cytometry. Blood *76*, 1739–1747.
11. Staupe, R.P., Lodge, K.E., Thambi, N., Toole, D., Tamburino, A.M., Chang, D., Howell, B.J., Hazuda, D.J., Vora, K.A., and Sullivan, N.L. (2022). Single cell multi-omic reference atlases of non-human primate immune tissues reveals CD102 as a biomarker for long-lived plasma cells. Commun. Biol. *5*, 1399.
12. Kreuser, S., Honaker, Y., Cheng, R.Y.-H., Dahl, N., Soligalla, R., Lopez, C., Rawlings, D.J., and James, R.G. (2021). Efficient methods for generation and expansion of, and gene delivery to Rhesus Macaque plasma B cells. Preprint at bioRxiv. https://doi.org/10.1101/2021.09.30.462595.
13. Duan, M., Nguyen, D.C., Joyner, C.J., Saney, C.L., Tipton, C.M., Andrews, J., Lonial, S., Kim, C., Hentenaar, I., Kosters, A., et al. (2023). Understanding heterogeneity of human bone marrow plasma cell maturation and survival pathways by single-cell analyses. Cell Rep. *42*, 112682.
14. Shaffer, A.L., Shapiro-Shelef, M., Iwakoshi, N.N., Lee, A.-H., Qian, S.-B., Zhao, H., Yu, X., Yang, L., Tan, B.K., Rosenwald, A., et al. (2004). XBP1, downstream of Blimp-1, expands the secretory apparatus and other organelles, and increases protein synthesis in plasma cell differentiation. Immunity *21*, 81–93.
15. Minnich, M., Tagoh, H., Bönelt, P., Axelsson, E., Fischer, M., Cebolla, B., Tarakhovsky, A., Nutt, S.L., Jaritz, M., and Busslinger, M. (2016). Multifunctional role of the transcription factor Blimp-1 in coordinating plasma cell differentiation. Nat. Immunol. *17*, 331–343.
16. Nutt, S.L., Hodgkin, P.D., Tarlinton, D.M., and Corcoran, L.M. (2015). The generation of antibody-secreting plasma cells. Nat. Rev. Immunol. *15*, 160–171.
17. Amanna, I.J., and Slifka, M.K. (2010). Mechanisms that determine plasma cell lifespan and the duration of humoral immunity. Immunol. Rev. *236*, 125–138.
18. Slifka, M.K., Antia, R., Whitmire, J.K., and Ahmed, R. (1998). Humoral immunity due to long-lived plasma cells. Immunity *8*, 363–372.
19. Bergen, V., Lange, M., Peidli, S., Wolf, F.A., and Theis, F.J. (2020). Generalizing RNA velocity to transient cell states through dynamical modeling. Nat. Biotechnol. *38*, 1408–1414.
20. Wolf, F.A., Hamey, F.K., Plass, M., Solana, J., Dahlin, J.S., Göttgens, B., Rajewsky, N., Simon, L., and Theis, F.J. (2019). PAGA: graph abstraction reconciles clustering with trajectory inference through a topology preserving map of single cells. Genome Biol. *20*, 59.
21. Horns, F., Vollmers, C., Croote, D., Mackey, S.F., Swan, G.E., Dekker, C.L., Davis, M.M., and Quake, S.R. (2016). Correction: Lineage tracing of human B cells reveals the in vivo landscape of human antibody class switching. Elife *5*, e23066. https://doi.org/10.7554/eLife.23066.
22. Zhang, F., Wang, L., Niu, X., Li, J., Luo, J., Feng, Y., Yang, Y., He, P., Fan, W., Liang, R., et al. (2019). Phenotypic Characterization of Chinese Rhesus Macaque Plasmablasts for Cloning Antigen-Specific Monoclonal Antibodies. Front. Immunol. *10*, 2426.
23. Meng, W., Li, L., Xiong, W., Fan, X., Deng, H., Bett, A.J., Chen, Z., Tang, A., Cox, K.S., Joyce, J.G., et al. (2015). Efficient generation of monoclonal antibodies from single rhesus macaque antibody secreting cells. mAbs *7*, 707–718.
24. Demberg, T., and Robert-Guroff, M. (2015). B-Cells and the Use of Non-Human Primates for Evaluation of HIV Vaccine Candidates. Curr. HIV Res. *13*, 462–478.
25. Hammarlund, E., Thomas, A., Amanna, I.J., Holden, L.A., Slayden, O.D., Park, B., Gao, L., and Slifka, M.K. (2017). Plasma cell survival in the absence of B cell memory. Nat. Commun. *8*, 1781.
26. Murphy, S.P., Choi, J.C., and Holtz, R. (2004). Regulation of major histocompatibility complex class II gene expression in trophoblast cells. Reprod. Biol. Endocrinol. *2*, 52.
27. Calame, K.L. (2001). Plasma cells: finding new light at the end of B cell development. Nat. Immunol. *2*, 1103–1108.
28. Silacci, P., Mottet, A., Steimle, V., Reith, W., and Mach, B. (1994). Developmental extinction of major histocompatibility complex class II gene expression in plasmocytes is mediated by silencing of the transactivator gene CIITA. J. Exp. Med. *180*, 1329–1336.
29. Garrido, F., Ruiz-Cabello, F., and Aptsiauri, N. (2017). Rejection versus escape: the tumor MHC dilemma. Cancer Immunol. Immunother. *66*, 259–271.
30. Aust, S., Felix, S., Auer, K., Bachmayr-Heyda, A., Kenner, L., Dekan, S., Meier, S.M., Gerner, C., Grimm, C., and Pils, D. (2017). Absence of PD-L1 on tumor cells is associated with reduced MHC I expression and PD-L1 expression increases in recurrent serous ovarian cancer. Sci. Rep. *7*, 42929.
31. Garrido, F. (2020). MHC Class-I Loss and Cancer Immune Escape (Springer International Publishing).
32. Bromage, E., Stephens, R., and Hassoun, L. (2009). The third dimension of ELISPOTs: quantifying antibody secretion from individual plasma cells. J. Immunol. Methods *346*, 75–79.
33. Balazs, A.B., Bloom, J.D., Hong, C.M., Rao, D.S., and Baltimore, D. (2013). Broad protection against influenza infection by vectored immunoprophylaxis in mice. Nat. Biotechnol. *31*, 647–652.
34. Wolf, F., Angerer, P., and Theis, F. (2018). SCANPY: large-scale single-cell gene expression data analysis. Genome Biol *19*, 15. https://doi.org/10.1186/s13059-017-1382-0.
35. La Manno, G., Soldatov, R., Zeisel, A., Braun, E., Hochgerner, H., Petukhov, V., Lidschreiber, K., Kastriti, M.E., Lönnerberg, P., Furlan, A., et al. (2018). RNA velocity of single cells. Nature *560*, 494–498. https://doi.org/10.1038/s41586-018-0414-6.

STAR★METHODS

KEY RESOURCES TABLE

REAGENT or RESOURCE	SOURCE	IDENTIFIER
Antibodies		
anti-CD3 APC-Cy7 clone:SP34-2	BD Bioscience	AB_396863
anti-CD3 A700 clone:SP34-2	BD Bioscience	AB_396938
anti-CD4 BV605 clone:L200	BD Bioscience	AB_2737833
anti-CD14 APC.Cy7 clone:MoP9	BD Bioscience	AB_396889
anti-CD20 PerCPCy5.5 clone:L27	BD Bioscience	340955
anti-CD31 BV605 clone:WM59	Biolegend	AB_2562149
anti-CD31 PE clone:WM59	BD Bioscience	AB_395839
anti-CD38 mFluor450 clone:OKT10	Caprico Biotechnologies	1008144
anti-CD38 FITC clone: OKT10	Caprico Biotechnologies	100815
anti-CD56 PE clone:MY31	BD Bioscience	347747
anti-CD138 PE clone:DL-101	Biolegend	AB_10900437
anti-CD59 APC clone:p282 (H19)	Biolegend	AB_2819929
anti-CD79A BV421 clone:HM47	BD Bioscience	AB_2737839
anti-IgG AF700 clone:G18-145	BD Bioscience	AB_10612406
anti-IgM FITC clone:G20-127	BD Bioscience	AB_396117
IgG capture antibody (ELISpot)	Invitrogen	A18813
IgG AP detection antibody (ELISpot)	Jackson	AB_2337601
Biological samples		
Frozen rhesus macaque (*macaca mulatta*) PBMC	BioIVT	–
Critical commercial assays		
NHP CD20 Isolation Kit	Miltenyi	130-091-105
IgG (Total) ELISA	Invitrogen	885055088
BAFF DuoSet ELISA	R&D Systems	DY124-05
Chromium Next GEM Single cell 3′ kit	10x genomics	PN-1000268
Chromium Next GEM Chip G Single cell 3′ kit	10x genomics	PN-1000127
Chemicals, peptides, and recombinant proteins		
IMDM	Gibco	12-440-053
StemCell B-cell supplement ACF	StemCell	10974
Multimeric CD40L	Adaptogen	AG-40B-0010
IL-2	PeproTech	AF-200-02
IL-10	PeproTech	200–10
IL-15	Miltenyi	130-095-765
IL-21	PeproTech	200–21
IL-6	PeproTech	200–06
IFN alpha 2B	Proteintech	HZ-1072
AF350 amine reactive dye	Invitrogen	A10168
Oligonucleotides		
CpG oligodeoxynucleotide 2006	Invitrogen	tlrl-2006-5
Deposited data		
scRNAseq raw and processed data	This paper	GEO: GSE247023
Data processing and analysis	This paper	https://doi.org/10.5281/zenodo.13737831

(*Continued on next page*)

Continued

REAGENT or RESOURCE	SOURCE	IDENTIFIER
Software and algorithms		
Cellranger	10X Genomics	https://support.10xgenomics.com/single-cell-gene-expression/software/downloads/3.0/
bcl2fastq2/2.20.0	illumina	https://support.illumina.com/downloads/bcl2fastq-conversion-software-v2-20.html
scanpy	Wolf et al. 2019[20,34]	https://scanpy.readthedocs.io/en/stable/
scVelo	Bergen et al., 2020[19]	https://scvelo.readthedocs.io/en/stable/
Velocyto	Manno et al., 2018[35]	http://velocyto.org/

METHOD DETAILS

B cell isolation

Frozen rhesus macaque (*macaca mulatta*) PBMCs were obtained from a commercial source (BioIVT). All source material was negative for the following viral antigens: HBV, SRV, SIV and STLV. The sex, genotype, and medical history of the animals was not provided, or considered in this study. Each data point was taken from a unique donor. In certain experiments, replicates were done using the same donor as indicated in the legends. Using an NHP specific CD20 positive selection isolation kit (Miltenyi Biotec), we isolated $CD20^+$ cells.

B cell culture conditions

Unless otherwise stated, all cells were cultured in a base media composed of Iscove's modified Dulbecco's medium (IMDM) (Gibco) supplemented with 10% FBS (Omega Scientific, FB-11), 1% Glutamax (Gibco), and 55 mM beta-mercaptoethanol (BMe). The commercial expansion cocktail we used was ImmunoCult-ACF Human B Cell Expansion Supplement (StemCell,10974) added to the base media as directed. For the defined B cell expansion and differentiation cocktails, the compositions are as follows. Phase I: 100 ng/mL recombinant human MEGACD40L (Enzo Life Sciences), 1 mg/mL CpG oligodeoxynucleotide 2006 (Invitrogen), 250 ng/mL IL-2 (PeproTech), 50 ng/mL IL-10 (PeproTech), and 10 ng/mL IL-15 (PeproTech), and 50 ng/mL IL-21 (PeproTech, 200-21). Phase II: IL-2 (250 ng/mL), IL-6 (50 ng/mL, PeproTech), IL-10 (50 ng/mL), and IL-15 (10 ng/mL). Phase III: IL-6 (50 ng/mL), IL-15 (10 ng/mL), and human interferon-a 2B (15 ng/mL, Sigma-Aldrich). All cell counts were done manually using a hemocytometer and trypan blue exclusion.

Flow cytometry

Flow cytometry was run on an LSR II flow cytometer (BD Biosciences) and data were analyzed using FlowJo software (Tree Star). All cells were fixed using Fix/Perm (BD Biosciences) for 20 min prior to intracellular staining and/or analysis. The flow cytometry antibody panels (Table S2) and gating schemes (Figure S1) are detailed in supplemental figures.

ELISpot and ELISAS

Membranes were coated with an anti-human IgG capture antibody (Thermo Fisher Scientific, A18813). Cells were then added to the plate in duplicate in 2-fold serial dilutions and incubated for 24hrs prior to detection using an alkaline phosphatase-linked anti-human IgG detection antibody (Jackson, 109-055-008). Protein secretion levels in culture supernatant were quantified via ELISA for human IgG (Invitrogen, 50-112-8849) and human BAFF (R&D Systems, DY124-05) using protocols recommended by the manufacturer.

Virus production and transduction

All viruses were made in house previously published methods. GFP-BAFF was previously published in King et al. AAV transduction was done on day three of culture. First cells were replated at $1.5x10^6$ cells/mL (unless otherwise noted) in IMDM + Bme + Glutamax + commercial supplement without FBS, then virus was added to culture at 20% by volume unless indicated otherwise and incubated at 37°C. After 2 h FBS was added back to the media for a final concentration of 10% and returned to 37°C. The next day the media volume was doubled in order to minimize any cell death caused by the addition of the AAV. For Lentivirus transduction, cells were replated at $1.5x10^6$ cells/mL IMDM alone, and virus was added. Cells were spinoculated for 30 min at RT at 400xg, then returned to 37°C for 6 h. After 6 h cells were spun down for 5 min at 400xg, half the media volume was taken, and IMDM was added back with two times the concentration of FBS, BMe, Glutamax, and commercial supplement and returned to 37°C.

Single cell RNA sequencing

NHP Single cell cDNA libraries were created using the 10x genomics platform (3′ chemistry V3.1 dual index kit, 10x genomics). Human cells were labeled with oligo-conjugated antibodies for tracking surface expression and sample identity labeling using the Biolegend Totalseq-B protocol. We prepared libraries using the 10X genomics platform (Single-Cell v3.1 with feature barcode dual index

kit, 10x genomics). Both NHP and human libraries were evaluated by tapestation (Agilent) before sequencing. Finally, libraries were sequenced with NextSeq 1000/2000 kit (Illumina).

Single-cell RNA-seq analysis

Fastq files were processed by CellRanger and Velocyto based on the customized *Macaca mulatta* gene annotation (Figure S2, Data S1). The h5 file is then further analyzed by a python script (https://github.com/Rene2718/Rhesus-Macaque-Plasma-B-Cells-single-cell-RNAseq-). Analysis including normalized, hierarchical clusterings, dimensional reduction, and cell clustering are analyzed by python package scanpy. Cell velocity and velocity pseudotime is analyzed by python package scVelo.

Comparison between monkey and human

Human and monkey's PCs are both generated *ex vivo*. For Human PCs scRNAseq experiment is generated by CITE-seq with surface markers oligos (IgM, CD38, and CD138). We used the surface markers to define human B cell subsets. For NHP cells, we fixed first with formaldehyde and recovered before running in 10X Chromium controller. We used transcriptomic clusters and markers to define the NHP B cell subsets. We categorized ActB, prePB, PB and PC, as well as different isotypes for the human and NHP datasets separately, and then compared the upregulated or downregulated genes between the species at different stages in PC differentiation as interpreted from the clustering.

QUANTIFICATION AND STATISTICAL ANALYSIS

Sample sizes in each experiment were described in each figure and legend. Flow cytometry, Elispot experiments are designed to have at least three biological replicates. scRNAseq experiment is designed to have two biological replicates. Clustering and gene differential analysis were using scanpy (1.8.2). Dataframe-based analysis and table output is generated by pandas (1.4.2). Other statistical analysis was performed by Graphpad (GraphPad, San Diego, CA). To assess significance for bar graph in Figures 1 and 7, we used two-sided unpaired t-test, significance is under a significance level of $\alpha = 0.05$. (*$p < 0.05$, **$p < 0.01$, ***$p < 0.001$). Data were presented as mean ± SD in all bar graph.

Cell Reports Methods, Volume *4*

Supplemental information

Generation, expansion, gene delivery, and single-cell profiling in rhesus macaque plasma B cells

Rene Yu-Hong Cheng, Anna E. Helmers, Shannon Kreuser, Noelle Dahl, Yuchi Honaker, Christina Lopez, David J. Rawlings, and Richard G. James

Supplemental

Table S1: Staining Panels. Related to Figures 1, 5, and 7.

**Panel 1: Day 0 Purity assessment**					
**Antigen**	Fluorophore	Clone	Vendor	Cat#	Dilution
**Live Dead**	AF350	N/A	Invitrogen	A10168	1:1000
**CD20**	PerCP-Cy5.5	L27	BD Bioscience	340955	1:20
**CD14**	APC-Cy7	MoP9	BD Bioscience	557831	1:200
**CD56**	PE	MY31	BD Bioscience	347747	1:200
**CD4**	BV605	L200	BD Bioscience	562843	1:200
**CD3**	A700	SP34	BD Bioscience	557917	1:200
**Panel 2: Phenotyping and transduction efficiency panel**					
**Antigen**	Fluor	Clone	Vendor	Cat#	Dilution
**Live Dead**	AF350	N/A	Invitrogen	A10168	1:1000
**CD20**	PerCP-Cy5.5	L27	BD Bioscience	340955	1:100
**CD14**	APC-Cy7	MoP9	BD Bioscience	557831	1:200
**CD31**	PE	WM59	BD Bioscience	555446	1:100
**CD38**	mFluor450	OKT10	Caprico Biotechnologies	1008144	1:100
**CD3**	AF700	SP34	BD Bioscience	557917	1:200
**Transduction**	GFP	--	---		---
**Panel 3: Phenotyping and intracellular Ig staining**					
**Antigen**	Fluor	Clone	Vendor	Cat#	Dilution
**Live Dead**	AF350	N/A	Invitrogen	A10168	1:1000
**CD20**	PerCP-Cy5.5	L27	BD Bioscience	340955	1:20
**CD14**	APC-Cy7	MoP9	BD Bioscience	557831	1:200
**CD138**	PE	DL-101	Biolegend	352306	1:100

**CD38**	mFluor450	OKT10	Caprico Biotechnologies	1008144	1:50
**CD38**	FITC	OKT10	Caprico Biotechnologies	100815	1:200
**CD3**	APC-Cy7	SP34	BD Bioscience	557917	1:200
**CD31**	BV605	WM59	Biolegend	303122	1:100
**CD59**	APC	p282	Biolegend	304712	1:50
**CD79A**	BV421	HM47	BD Bioscience	BDB566225	1:200
**IgG-ic**	AF700	G18-145	BD Bioscience	561296	1:200
**IgM-ic**	FITC	G20-127	BD Bioscience	555782	1:200

Table S2: AAV transduction with different titers. Related to Figure 7.

		DAY 5		
		698	A17104	relative titer
	no AAV	1.55	0.58	0
**1**	scAAV1	9.41	2.33	0.664063
**2**	AAV2	18.4	9.41	1
**3**	AAV2.5	11.2	6.36	0.46875
**4**	scAAV6	9.46	2.09	0.086328
**5**	AAVD-J	22	9.92	1.757813
**6**	AAV5	28.4	12.3	4.84375
**7**	AAV8	7.48	1.75	2.402344
**8**	AAV9	3.96	0.99	2.773438
**9**	AAVRh.8	1.1	0.36	0.332031
**10**	AAV11cap11	0.91	0.34	0.214844
**11**	AAVcap11	0.94	0.4	0.46875
**12**	AAV HSC7	1.8	0.59	0.992188
**13**	AAV1110 pLT AAV Help (AAV2) Y444F, Y500F, Y730F	16.2	6.95	0.5
		DAY 7		
		698	A17104	relative titer
	no AAV	2.42	1.05	0
**1**	scAAV1	5.91	2.34	0.664063
**2**	AAV2	26.5	23.9	1
**3**	AAV2.5	14.6	11.3	0.46875
**4**	scAAV6	6.74	1.82	0.086328
**5**	AAVD-J	33.7	23.2	1.757813
**6**	AAV5	26.2	14.5	4.84375
**7**	AAV8	5.18	2.18	2.402344
**8**	AAV9	3.2	1.9	2.773438

**13**	AAV1110 pLT AAV Help (AAV2) Y444F, Y500F, Y730F	19	13.7	0.332031
		DAY 10		
		698	A17104	relative titer
	no AAV	7.27	1.63	0
**1**	scAAV1	3.28	1.37	0.664063
**2**	AAV2	21.5	21.1	1
**3**	AAV2.5	10.4	8.34	0.46875
**4**	scAAV6	3.78	1.3	0.086328
**5**	AAVD-J	35.6	26.4	1.757813
**6**	AAV5	22.9	14.5	4.84375
**7**	AAV8	4.27	2.11	2.402344
**8**	AAV9	2.92	1.58	2.773438
**13**	AAV1110 pLT AAV Help (AAV2) Y444F, Y500F, Y730F	12.5	10.5	0.332031

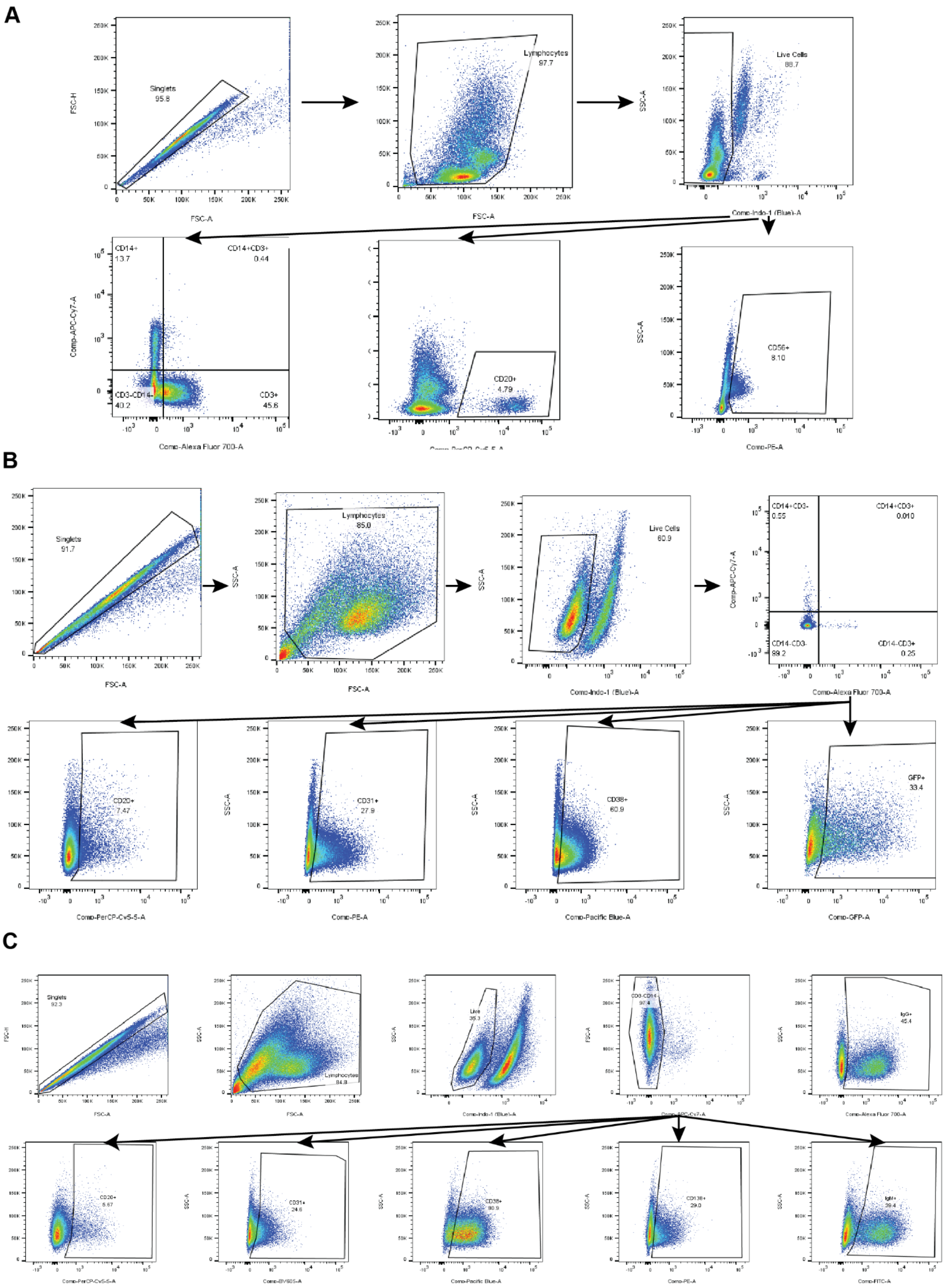

A
Singlets
95.8
Lymphocytes
97.7
Live Cells
88.7
FSC-H
FSC-A
SSC-A
Comp-Indo-1 (Blue)-A
CD14+
13.7
CD14+CD3+
0.44
CD3-CD14-
40.2
CD3+
45.6
Comp-APC-Cy7-A
Comp-Alexa Fluor 700-A
CD20+
4.79
CD56+
8.10
Comp-PE-A
B
Singlets
91.7
Lymphocytes
85.0
Live Cells
60.9
CD14+CD3-
0.55
CD14+CD3+
0.010
CD14-CD3-
99.2
CD14-CD3+
0.25
CD20+
7.47
CD31+
27.9
CD38+
60.9
GFP+
33.4
Comp-PerCP-Cy5-5-A
Comp-PE-A
Comp-Pacific Blue-A
Comp-GFP-A
C
Singlets
92.3
Lymphocytes
84.8
Live
35.3
CD3-CD14-
97.4
IgG+
45.4
Comp-Alexa Fluor 700-A
CD20+
5.67
CD31+
24.6
CD38+
80.9
CD138+
29.0
IgM+
39.4
Comp-PerCP-Cy5-5-A
Comp-BV605-A
Comp-Pacific Blue-A
Comp-PE-A
Comp-FITC-A

Figure S1. Flow cytometry gating strategy. Related to Figures 1, 5, and 7. A. Gating scheme for flow cytometry Panel 1 (Table S1) to determine purity at the time of isolation. **B.** Gating scheme for flow cytometry Panel 2 (Table S1) to determine B cell phenotype and transduction efficiency through GFP expression. **C.** Gating scheme for flow cytometry Panel 3 (Table S1) to determine B cell phenotype and intracellular Ig production.

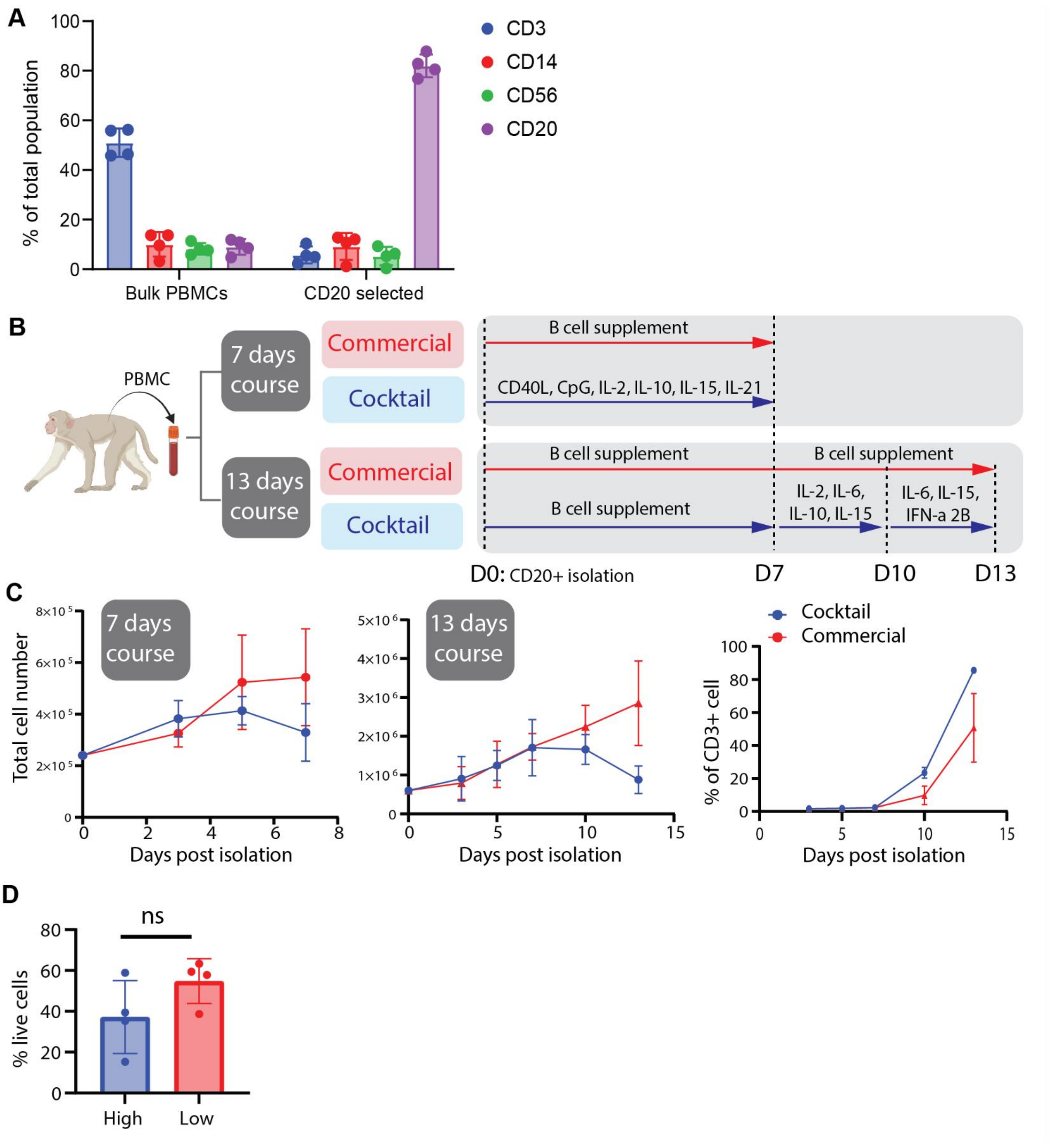

Figure S2. Method of ex vivo expansion and differentiated NHP PCs. Related to Figure 1. A. Following isolation of CD20+ Rhesus B cells from PBMCs, we assessed cell purity via flow cytometry using the indicated antibodies (4 donors, n=4). **B.** Schematic workflow of monkey *ex vivo* differentiated PC generation with two different time course and two different conditions. **C.** CD20+ NHP B cells were cultured for 7 days/13 days with defined cocktail or commercial expansion medium (1.5-1x10^6 cells/mL) (3 donors, n=3), Total cells were counted on days 3, 5, and 7 (left and middle). Percentage of contaminating

T cells cells (CD3+, left). **D.** To assess the impact of cell density, CD20+ NHP B cells were cultured in the commercial B cell medium for 7 days (Plated and maintained 1.5x10^{6} cells/mL versus plated at 2.5x10^{5} cells/mL and maintained at 1x10^{5} cells/mL; 4 donors, n=3). At day 7 following isolation, the percent viability was analyzed using flow cytometry. Data presented as individual data points and error bar represent standard deviation.

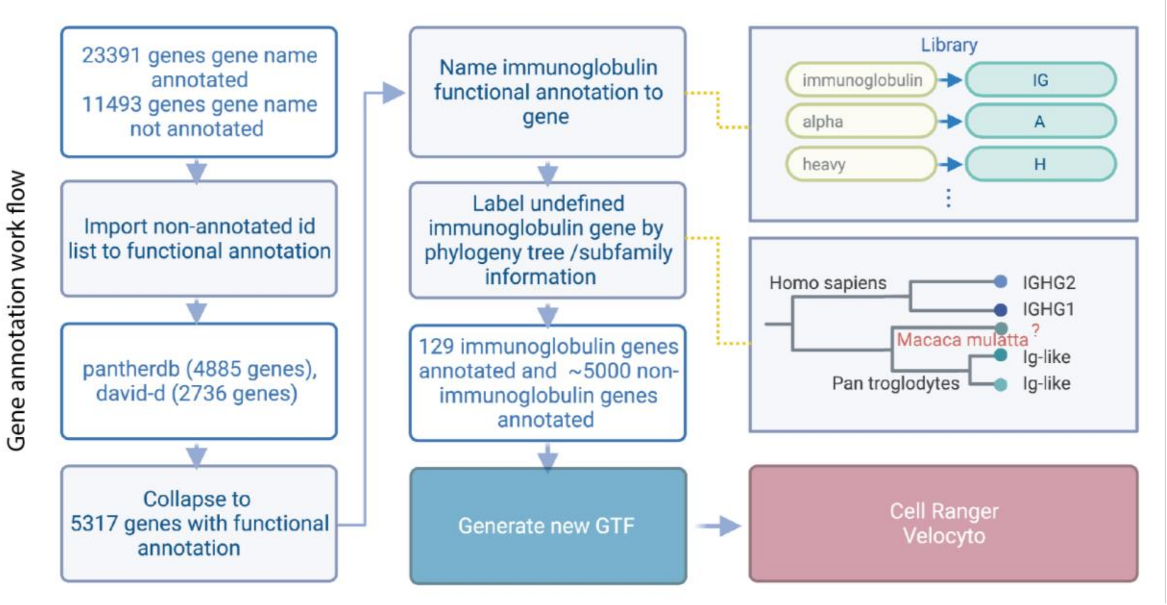

Figure S3. Monkey (Macaca mulatta) gene annotation workflow. Related to Figure 1. Gene annotation workflow. (GTF: Gene transfer fomat, Macaca_mulatta.Mmul_10.104.chr.gtf, generated new GTF: genes.gtf)

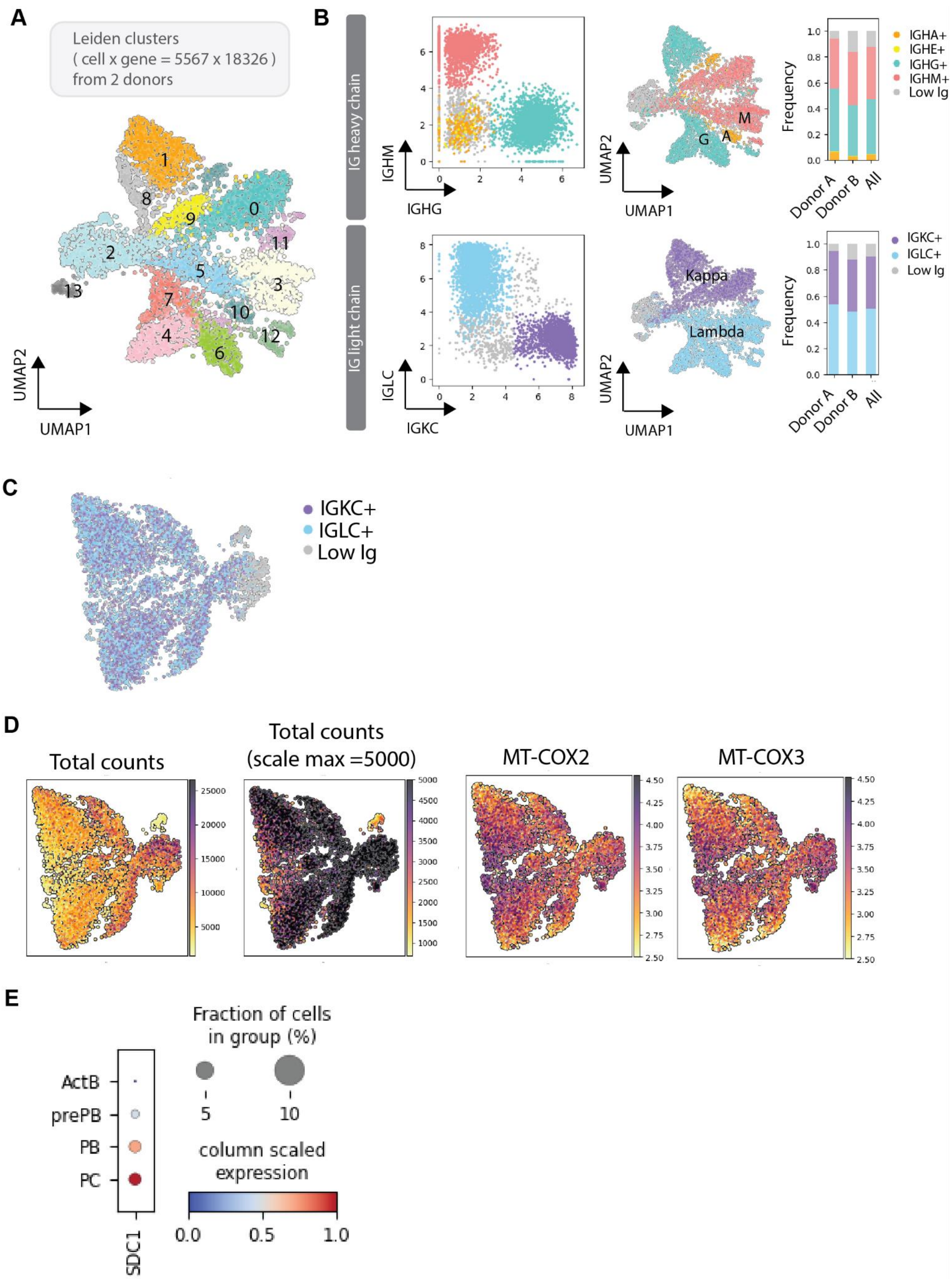

A
Leiden clusters
(cell x gene = 5567 x 18326)
from 2 donors
0
1
2
3
4
5
6
7
8
9
10
11
12
13
UMAP2
UMAP1
B
IG heavy chain
IGHM
IGHG
UMAP2
UMAP1
M
G
A
Frequency
Donor A
Donor B
All
IGHA+
IGHE+
IGHG+
IGHM+
Low Ig
IG light chain
IGLC
IGKC
Kappa
Lambda
IGKC+
IGLC+
Low Ig
C
IGKC+
IGLC+
Low Ig
D
Total counts
Total counts
(scale max =5000)
MT-COX2
MT-COX3
E
Fraction of cells
in group (%)
5
10
column scaled
expression
0.0
0.5
1.0
ActB
prePB
PB
PC
SDC1

Figure S4. Monkey *ex vivo* differentiated PCs subsets classification and differential expression analysis for PCs. Related to Figures 1, 2, and 3. A. Single cell graph UMAP dimension-reduction projection of day13 B cells (n = 5567) from two biological replicates. **B.** Classification of immunoglobulin heavy chain (upper panel) and light chain (lower panel). UMAP projection of the subclass of IGHG+, IGHA+, IGHM+ cells (upper panel) and IGKC+, IGLC+ cells (lower panel), and bar graph represent the percentage of subclasses from two individual donor. **C.** UMAP projection of IGKC+, IGLC+ cells in LC-independent clusters. **D.** Heatmap showing total count and expression of representative MT genes in LC-independent clusters. **E.** Dotplot visualization of *ex vivo* differentiated monkey PCs: subsets are listed on y-axis and SDC1 (CD138) gene on x-axis.

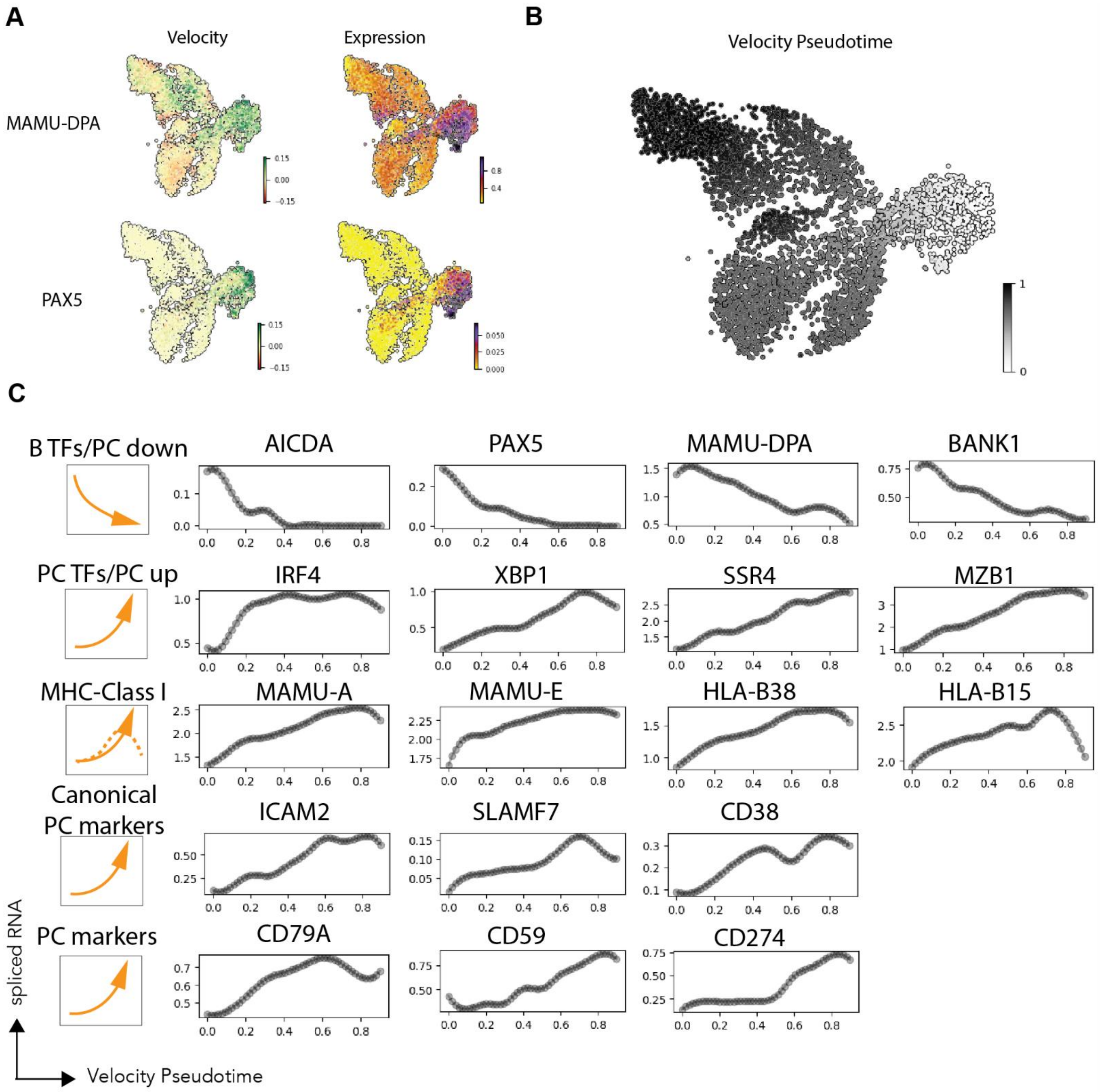

D

	fit_diff_kinetics	fit_pval_kinetics
**EZH2**	ActB	1.70e-08
**TUBA1A**	ActB	1.97e-06
**BIRC3**	prePB	1.08e-03
**MAN1A1**	mPC	1.56e-04
**FUT8**	aPC	8.95e-03
**FNDC3A**	aPC	6.73e-04
**IGF1**	gPC	3.10e-03
**CD274**	gPC	2.30e-06

Figure S5. RNA velocity of immunoglobulin and kinetic differentiation analysis. Related to Figure 4. A. Heatmap of indicated gene velocity and RNA expression level superimposed on to UMAP. **B.** Heatmap of velocity pseudotime superimposed on to UMAP. **C.** Scatter plot of indicated genes with velocity pseudotime and mean of spliced mRNA in every 0.1 pseudotime. **D.** scVelo kinetic differentiation analysis.

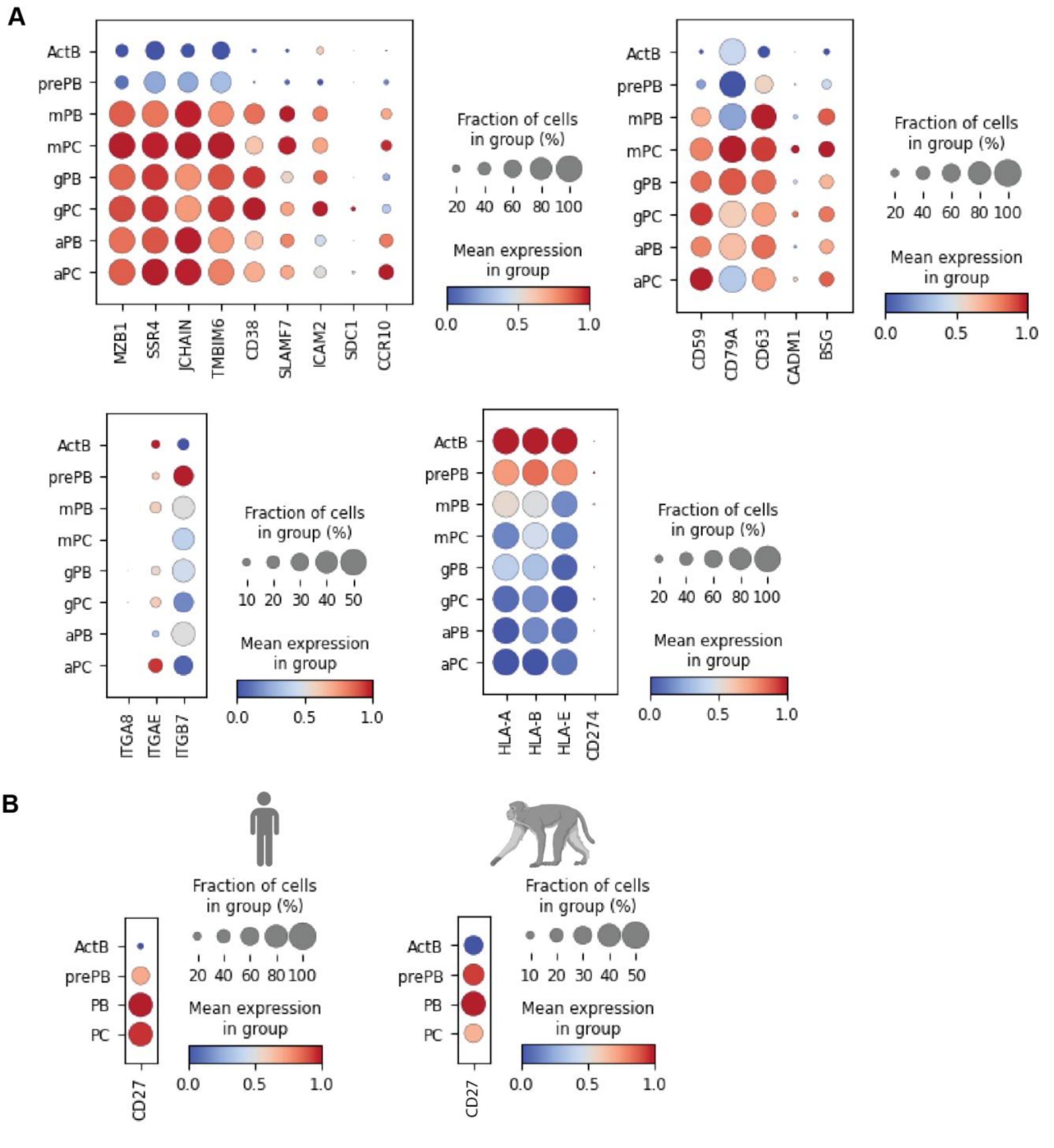

Figure S6. Comparison of human and monkey PC makers by scRNAseq analysis. Related to Figure 6. A. Dotplot visualization of *ex vivo* differentiated human PC: subsets with different isotypes are listed on y-axis and representative genes from Fig. 3D are listed along the x-axis. **B.** Dotplot visualization of *ex vivo* differentiated human and monkey PC: subsets are listed on y-axis and CD27 gene on x-axis.

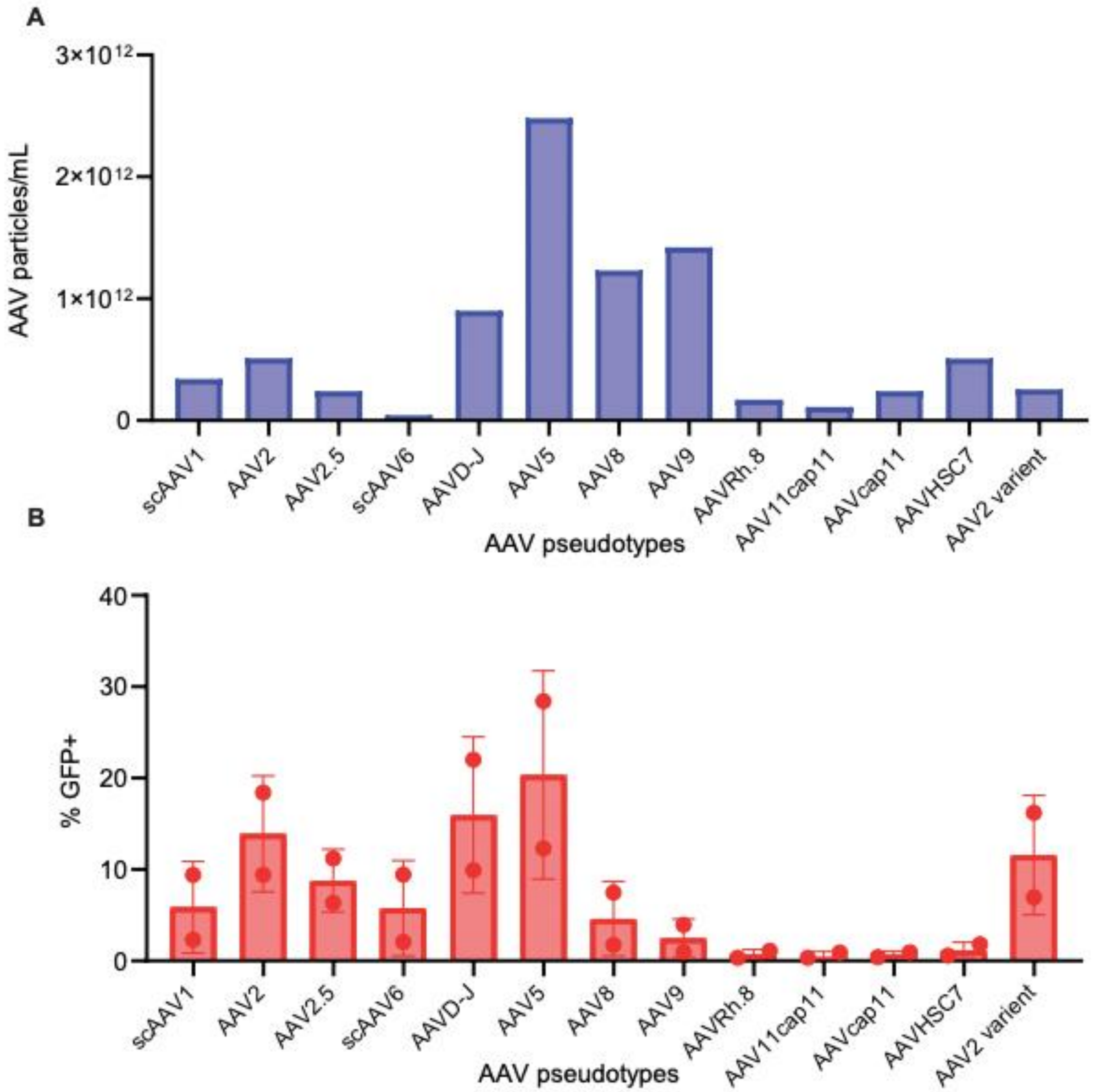

Figure S7. NHP B cell transduction with AAV. Related to Figure 5. A-B NHP B cells were transduced on day 3 of culture. 20% by volume was added of GFP encapsulated in different AAV pseudotypes, with varying titers **A.** and flow for GFP+ **B.** was run 2 days later.
